# Targeting Wnt/**β**-catenin and circadian regulator restores PRC2/EZH2-controlled chromatin bivalency and suppresses cell state diversity

**DOI:** 10.1172/JCI200260

**Published:** 2026-03-17

**Authors:** Yatian Yang, Xiong Zhang, Varadha Balaji Venkadakrishnan, Hongye Zou, Xingling Zheng, Shiyao Guo, Christopher Z. Chen, Alexander D. Borowsky, Eva Corey, Ronald M. Evans, Allen C. Gao, Marc A. Dall’Era, Amina Zoubeidi, Primo N. Lara, Hsing-Jien Kung, Xinbin Chen, Himisha Beltran, Hong-Wu Chen

**Affiliations:** 1 Department of Biochemistry and Molecular Medicine, School of Medicine, UCD, Sacramento, California, USA.; 2 Department of Medical Oncology, Dana-Farber Cancer Institute, Boston, Massachusetts, USA.; 3 Harvard Medical School, Boston, Massachusetts, USA.; 4 Pharmaceutical Sciences and Pharmacogenomics (PSPG) Graduate Program, UCSF, San Francisco, California, USA.; 5 Department of Pathology and Laboratory Medicine, School of Medicine, UCD, Sacramento, California, USA.; 6 Department of Urology, University of Washington, Seattle, Washington, USA.; 7 Gene Expression Laboratory, Salk Institute for Biological Studies, La Jolla, California, USA.; 8 Department of Urologic Surgery, School of Medicine, UCD, Sacramento, California, USA.; 9 Department of Urologic Sciences, University of British Columbia, Vancouver, British Columbia, Canada.; 10 Vancouver Prostate Centre, Vancouver, British Columbia, Canada.; 11 Division of Hematology Oncology, Department of internal medicine, and; 12 Comprehensive Cancer Center, UCD, Sacramento, California, USA.; 13 Department of Surgical and Radiological Sciences, UCD, Davis, California, USA.; 14 US Veterans Affairs Northern California Health Care System-Mather, Mather, California, USA.

**Keywords:** Cell biology, Genetics, Oncology, Epigenetics, Molecular biology, Prostate cancer

## Abstract

PRC2/EZH2 inhibitors (PRC2i/EZH2i) are promising for the treatment of advanced cancers including metastatic prostate cancer. Here, we show that PRC2i/EZH2i alone or in combination with androgen receptor (AR) inhibitors induced diverse cell state programs (CSPs) (e.g., response to stress or IFN, MYC targets, stem cells, EMT lineage plasticity, and multiple developmental programs), which led to increased tumor cell invasion, metastasis, and resistance to other drugs, in addition to modest suppression of tumor growth. In contrast to the current perception, our comprehensive, integrated genomics and epigenomics profiling of patient-derived xenografts (PDXs) and clinical tumors revealed that PRC2/EZH2 suppressed CSP genes by maintaining chromatin bivalency. Hyperactive Wnt/β-catenin signaling and inhibitors of polycomb-repressive complex 2/enhancer of zeste homolog 2 (PRC2/EZH2) and the AR alter chromatin bivalency through antagonism of PRC2 and stimulation of MLL2/KMT2B in a feed-forward manner. The circadian rhythm regulator REV-ERBα unexpectedly reprogrammed β-catenin in promoting bivalency resolution and CSP gene expression. Dual targeting of Wnt/β-catenin and EZH2 diminished diverse cell states by restoring bivalency and effectively blocked tumor growth. Our findings provide unexpected insights into chromatin bivalency and dysregulated circadian rhythms in the control of cell state diversity and identify alternative therapeutic strategies that target PRC2/EZH2 for advanced malignancies.

## Abstract 2

Targeting EZH2 in advanced prostate cancer may unintentionally promote aggressive cell states, but combination therapies can prevent tumor spread and resistance.

## Graphical abstract

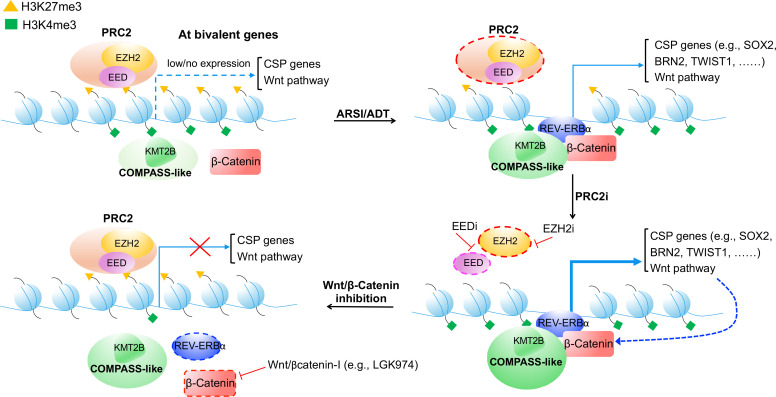

## Introduction

The polycomb-repressive complex 2 (PRC2) is a major epigenetic writer of the gene-silencing mark H3K27me3, with enhancer of zeste homolog 2 (EZH2) being its catalytic subunit and SUZ12, EED, and RBBP4/7 its core subunits (1, 2). During embryonic development, the PRC2 complex plays an important role in cell-fate transitions and differentiation through silencing of lineage-specific transcription factors (TFs) and other lineage-related gene programs. Most of those genes are comarked at the promoters with both transcriptionally active H3K4me3 and repressive H3K27me3 marks which are defined as chromatin bivalency and are “written” primarily by the methylases KMT2B (for H3K4me3) and EZH2/PRC2 (3, 4). Loss of PRC2 function leads to bivalency resolution (i.e., diminished H3K27me3 and/or increased H3K4me3) and, consequently, induction of genes critical for embryonic stem cell (ESC) differentiation to diverse cell lineages (5).

In cancer, *EZH2* is often overexpressed, and its overexpression is generally associated with disease progression. Some of the well-known PRC2 targets include cell-cycle regulator genes such as *CDKN1A*/p21 and *CDKN2A*/p16 and developmental gene programs (3, 4). In addition to its canonical functions described above, EZH2 also displays a noncanonical transcriptional coactivator function. In prostate adenocarcinoma, EZH2 also acts as an androgen receptor (AR) coactivator to stimulate the expression of a subset of AR target genes (6–8). Currently, drugs targeting PRC2/EZH2 have been on clinical trials in different cancers including advanced prostate cancer (PCa). However, only a fraction of patients showed a response, albeit it a limited one. In specific preclinical models, high antitumor efficacy of EZH2 inhibitors can only be achieved in combination with other therapeutics (9, 10). Therefore, there is an urgent need to better understand the mechanism underlying the response and resistance to drugs targeting PRC2 and EZH2.

Diverse cell states in tumors defined by scRNA-seq profiling reveal tumor-heterogeneous expression of a partial version of developmental, regulatory, and physiological gene programs or cell state programs (CSPs) (11, 12). Those CSPs are often enriched in genes involved in the response to stress and specification of diverse cell lineages (12). Cell state diversity including lineage plasticity (LP) is now recognized as a major source of tumor heterogeneity and a key mechanism of cancer progression and therapy resistance (13). Prolonged treatment of PCa with AR signaling inhibitors (ARSIs) transforms subpopulations of adenocarcinoma cells to variants including neuroendocrine PCa (NEPC) cells, which express NE lineage markers and neurogenesis programs, and other cells with induced EMT and stemness programs (14, 15).

Although epigenetic mechanisms are perceived as the main driving force of cell state diversity (16, 17), how the diversity initiates at the chromatin level is poorly understood. Previous studies using murine genetic models implicated a potential promotional function of EZH2 in the LP of PCa (13). Our recent study demonstrated that EZH2 inhibition alters chromatin bivalency and induces specific NE lineage TF expression in patient-derived organoid models of de novo NEPC (18). However, the mechanism of how chromatin bivalency plays a role in the control of cell state diversity and cell-fate commitment in cancer is still poorly understood. In particular, whether inhibition of PRC2/EZH2 in combination with ARSI drugs promotes or inhibits cell state diversity is unclear.

We performed sequential ChIP-seq, which identified approximately 3,000–5,000 bivalent promoters in PCa tumors, encompassing CSP genes. Our further studies revealed a crucial function of PRC2/EZH2 in maintenance of the bivalency and suppression of tumor cell–state diversity. Dual targeting of PRC2/EZH2 and the AR dramatically induced CSPs and increased tumor metastasis and drug resistance. Unexpectedly, we found that hyperactive Wnt/β-catenin signaling and the circadian rhythm regulator REV-ERBα drove bivalency resolution and facilitated the emergence of diverse cell states via a feed-forward mechanism involving both EZH2 inhibition and *KMT2B* activation. Importantly, dual inhibition of EZH2 and Wnt/β-catenin restored chromatin bivalency and effectively suppressed cell state plasticity and tumor growth. Our findings highlight a crucial role of chromatin bivalency in tumor heterogeneity and the unintended effects from drugs targeting PRC2/EZH2 and offer strategies for more effective epigenome targeting.

## Results

### Therapeutic targeting of PRC2/EZH2 and AR increases drug resistance and promotes tumor metastasis.

**Figure 1:**
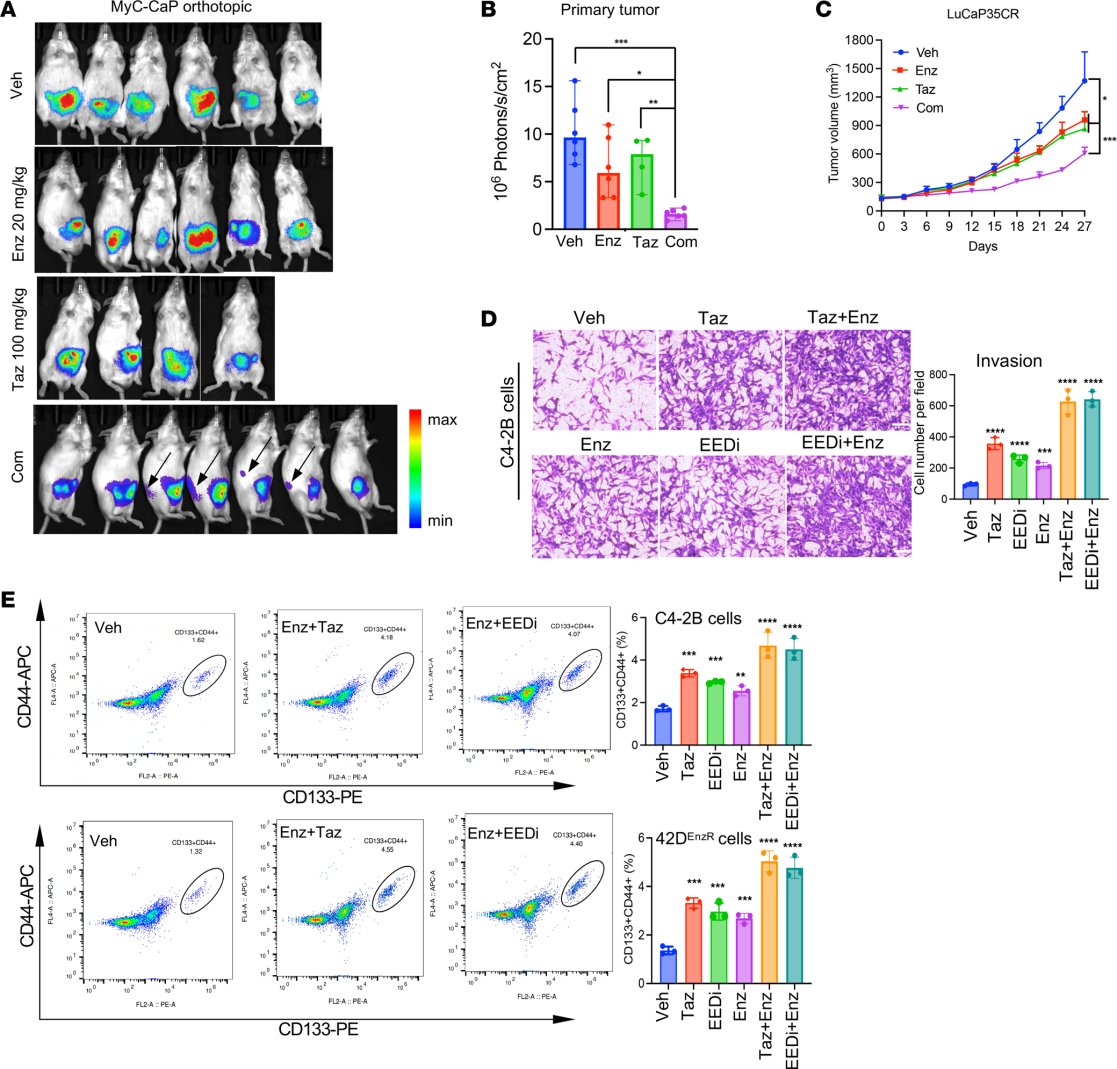
Therapeutic targeting of PRC2/EZH2 and the AR increases drug resistance and promotes tumor metastasis. (**A**) Bioluminescence imaging of luciferase-expressing Myc-CaP tumors implanted orthotopically into mice treated with the indicated drugs for 14 days. Arrows indicate liver metastasis in mice with the combined treatment. Images shown are representative of 6 mice per group. (**B**) Bar graphs of bioluminescence quantification of primary tumors. *P < 0.05, **P < 0.01, and *** *P* < 0.001, by 2-tailed Student’s *t* test. (**C**) Growth curves of LuCaP35CR PDX tumors in mice treated with vehicle or the indicated compounds for the indicated durations, 7 times per week (*n* = 5 mice per group). **P < 0.01 and ***P < 0.001, by 2-tailed Student’s *t* test. (**D**) Representative images of C4-2B cells invaded through Matrigel and stained with crystal violet and treated as indicated for 48 hours. Images are representative of 3 independent experiments. Scale bar: 50μM. (**E**) Flow cytometric analysis of CD44 and CD133 markers in C4-2B and 42D^EnzR^ cells treated with the indicated compounds for 48 hours. **P < 0.01 and ***P < 0.001, by 2-tailed Student’s *t* test. Veh, vehicle.

To better understand the efficacy of combined treatments with PRC2 inhibitors (PRC2i) and ARSIs, we first measured the effects on cell viability and tumor growth by the EZH2 inhibitors tazemetostat/EPZ6438 (Taz) and mevrometostat/PF-06821497 (Mev) (9), the EED inhibitor EED226 (EEDi) and the AR antagonist enzalutamide (Enz) in different PCa models. We chose those agents for their high target selectivity and clinical relevance. With the exception of EED226, Taz and Mev are being used for treating patients with cancer or in clinical trials (9). Consistent with previous findings of the noncanonical role of EZH2 as an AR coactivator (6–8), the PRC2i displayed more pronounced growth inhibition in ARSI-sensitive LNCaP and Myc-CaP cells than the resistant cells (C4-2B and 42D^EnzR^) (Supplemental Figure 1A; supplemental material available online with this article; https://doi.org/10.1172/JCI200260DS1). Notably, Taz and its combination with Enz did not completely block tumor growth, as significant tumor growth persisted in both the syngeneic and patient-derived xenograft (PDX) models (Figure 1, A–C). Importantly, the combined treatment markedly stimulated tumor metastasis to the liver (Figures 1A and Supplemental Figure 1B).

The unexpected effects of cotargeting PRC2 and the AR on tumor metastasis prompted us to further examine the consequences on tumor cell aggressiveness. Treatment of cells with inhibitors of EZH2 or EED alone significantly increased cancer stem cell (CSC) populations detected by tumor sphere formation and flow cytometric analysis of CSC markers. The treatment also strongly enhanced cell activities of migration and invasion. The combined treatments with Enz elicited more pronounced effects (Figures 1, D and E, and Supplemental Figure 1, C and D). These results were consistent with the prometastatic effects seen with the combined treatments. Moreover, treatments with the PRC2i alone or in combination with Enz significantly increased cell survival (Supplemental Figure 1E). Strikingly, they also markedly increased the IC_50_ values of targeted therapy drugs such as poly (ADP-ribose) polymerase inhibitor (PARPi) (niraparib and rucaparib, 50-fold increase from 0.07 for niraparib and 100-fold increase from 0.14 for rucaparib in LNCaP cells), Akt kinase inhibitor (capivasertib, 6-fold increase from 1.6 in LNCaP cells), Aurora kinase A inhibitor (alisertib, 5-fold increase from 6.63 in LNCaP cells), and receptor tyrosine kinase inhibitor (cabozantinib, 7-fold increase from 24.09 in LNCaP cells), as well as chemotherapeutic drugs (cabazitaxel and docetaxel, 2,800-fold increase from 0.0002 for cabazitaxel and 100-fold increase from 0.29 for docetaxel) (Supplemental Figure 1F). Taken together, the above results suggest that inhibition of PRC2, particularly in combination with ARSIs, strongly increased tumor stemness, invasion, drug resistance, and metastasis.

### Therapeutic targeting of PRC2/EZH2 and the AR promotes cell state diversity.

**Figure 2:**
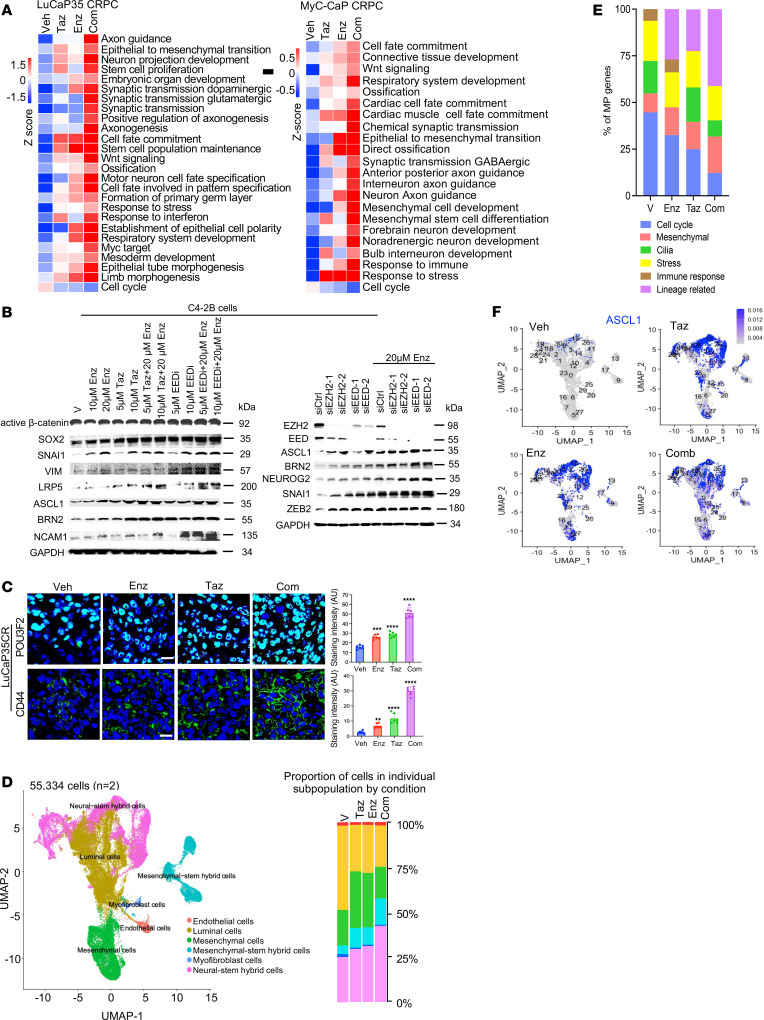
Therapeutic targeting of PRC2/EZH2 and the AR promotes cell state diversity. (**A**) Heatmaps of the gene set variation analysis (GSVA) score of the indicated programs detected by RNA-seq in LuCaP35CR and MyC-CaP CR tumors in mice treated as indicated for 10 days. (**B**) Western blots of the indicated proteins in C4-2B cells treated with the indicated compounds or siRNAs for 72 hours. (**C**) Representative confocal microscopy images of LP protein expression in LuCaP35CR tumors from mice treated as indicated. Images shown are representative of 5 mice per group. Scale bars: 50 μm. Bar graphs display ImageJ-analyzed IF intensity from 7 randomly selected fields per tumor. **P < 0.01, ***P < 0.001, and ****P < 0.0001, by 2-tailed Student’s *t* test. (**D**) Uniform manifold approximation and projection (UMAP) visualization of different types of cells from scRNA-seq in LuCaP35CR tumors from mice treated as indicated for 10 days (left). Proportion of cells in individual subpopulations by treatment (right). (**E**) Bar graphs display the proportion of genes in the 6 different MPs in scRNA-seq data from LuCaP35CR tumors from mice treated as indicated. (**F**) UMAP visualization of the different types of epithelial cells colored by a gradient of *ASCL1* expression. The minimum score is indicated in light gray, and the maximum score is indicated in blue. Com, combination.

To understand the mechanism underlying the effects by dual targeting of PRC2/EZH2 and the AR, we first performed bulk RNA-seq analysis of tumors and cells treated with PRC2i alone or in combination with Enz. Consistent with the growth inhibition effects, PRC2i treatments markedly downregulated the expression of cell-proliferation/cell-cycle genes and programs including cyclins and cyclin-dependent kinases (CDKs) (e.g., *CCND3*, *CCNE2*, *CDK2*, and *CDK4*) (Figure 2A and Supplemental Figure 2, A and B). Known PRC2 target genes such as *CDKN1A* and *CDKN2B* were induced. However, other cell-cycle genes (e.g., *CDK6*, *CCND2*, *CCNA1*, *CDC2*/*CDK1*) were also upregulated (Supplemental Figure 2C). In line with previous studies (7, 8), the inhibitors also downregulated the expression of a set of genes that were previously reported to be common targets of EZH2 and the AR (Supplemental Figure 2D).

Remarkably, the treatments also induced programs of development of diverse tissues or organs (e.g., nervous system, respiratory, mesenchyme/connective), cell-fate commitment, stem cell differentiation, epithelial-mesenchymal transition (EMT), response to stress, hypoxia or IFN, MYC targets, and Wnt signaling. Combination treatment with Enz resulted in further marked stimulation of the expression of those programs (Figure 2A and Supplemental Figure 2E). Notably, gene expression of major LP driver TFs (e.g., *ASCL1*, *POU3F2*, *SOX2*, and *ZEB1*) and Wnt signaling (e.g., *WNT9A*/*10A*, *LRP5*, *DVL1*/*2*) were synergistically induced (Figure 2B and Supplemental Figure 2F). Moreover, knockdown of EZH2 and EED alone or in combination with ARSIs strongly induced protein expression of the CSP drivers and markers (19) (Figure 2B and Supplemental Figure 2G). Immunofluorescence (IF) analysis of tumors also demonstrated that major CSP drivers and markers were synergistically increased (Figure 2C and Supplemental Figure 2H).

Since-cell state diversity is a major mechanism of cancer progression and resistance (13), we wondered whether the treatment-induced gene expression reprogramming was related to changes in cell state diversity or CSP. To examine the potential effects on cell states, we performed scRNA-seq analysis of PDX tumors treated with the inhibitors. Using an unsupervised strategy and cell lineage markers curated previously (20–24), we generated 32 cell clusters and classified them into 6 subpopulations: luminal epithelial cells, mesenchymal cells, mesenchymal or neural stem hybrid cells, which highly expressed both mesenchymal or neural markers and stem markers, myofibroblasts, and endothelial cells (Figure 2D). Significantly, either Taz or Enz alone increased the subpopulations of mesenchymal-stem hybrid cells and mesenchymal cells and decreased luminal cell populations. Remarkably, the Taz and Enz combination strongly increased neural stem hybrid cells (from 25% to 43.1%) and mesenchymal-stem hybrid cells (from 4.8% to 15%) and further decreased luminal cell percentages (from 46.8% to 22.6%) (Figure 2D).

Recent analyses of scRNA-seq data from clinical tumors revealed that cell state diversity is displayed by heterogenous expression of a number of gene programs, in addition to cell lineage–related ones. Thus, using non-negative matrix factorization (NMF) analysis (12) of scRNA-seq data from 50 different clinical PCa tumors (21, 25–28), we identified 1,209 cell state genes in 35 meta-programs (MPs) in the PCa tumors that belong to 6 functional Gene Ontology (GO) annotations (e.g., cell-cycle, mesenchymal, and lineage related) (Supplemental Figure 2I). Moreover, our NMF analysis revealed that Taz and/or Enz increased the number of lineage-related MPs genes and reduced the number of genes in cell-cycle MPs (Figure 2E).

Further analysis revealed that the combination of Taz and Enz induced the expression of diverse lineage-related genes in a subset of the tumor cells. For instance, a subset of neural-stem hybrid cells also expressed drivers or markers of respiratory development (e.g., *HOPX* and *SFTPA2*) (29), bone ossification (e.g., *BMP3*/*4* and *ALPL*), or connective tissue development (e.g., *HOXA11*, and *GREM1*) (30, 31), in addition to the known tumor LP genes (e.g., *ASCL1*, *SOX2*, *CHGA*) (32, 33) (Figure 2F and Supplemental Figure 2, J and K). A subset of mesenchymal-stem hybrid cells also expressed genes of ossification or connective tissue development (Supplemental Figure 2K). Strikingly, a subset of luminal cells also expressed genes involved in diverse tissue development (e.g., *HOPX*, *RFLNA*, *ALPL*, *BARX2*) as well as in stemness and NE features (Supplemental Figure 2K). Together, these results strongly suggest that targeting EZH2 and the AR alone or in combination promoted cell state diversity through induction of a mixed set of CSP genes and by increasing the number of cells with hybrid states.

### Induction of cell state diversity is associated with the alteration of chromatin bivalency.

**Figure 3:**
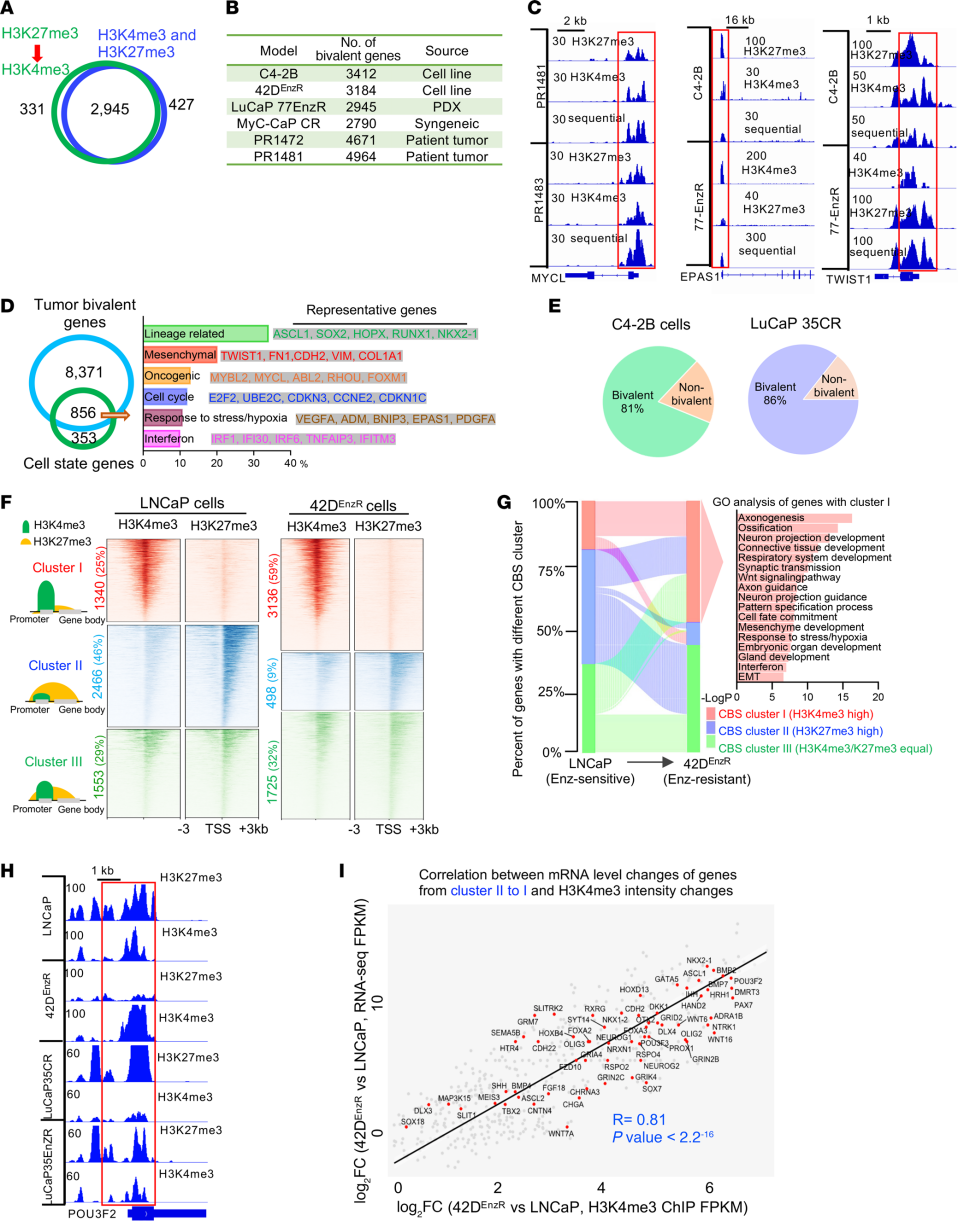
Induction of cell state diversity is associated with alteration of chromatin bivalency. (**A**) Venn diagram of overlapping genes (*n* = 2,945) among 3,276 genes, with peaks detected by H3K27me3 to H3K4me3 sequential ChIP-seq (green) and among 3,372 genes, with peaks detected by H3K4me3 and H3K27me3 separate ChIP-seq (blue) in LuCaP77EnzR PDX tumors. (**B**) Number of bivalent genes identified in the indicated models and in fresh human tumors. (**C**) Integrative Genomic Viewer (IGV) snapshots of sequential or separate ChIP-seq of H3K27me3 and H3K4me3 at representative CSP genes in C4-2B cells, LuCaP77EnzR PDX tumors, and fresh human tumors. (**D**) Venn diagram of overlapping genes (*n* = 856) among bivalent genes in all models and cell state genes and bar plot of major programs from GO analysis. (**E**) Pie charts of the percentage of bivalent and nonbivalent genes upregulated by Taz and Enz combination treatment in the indicated models. (**F**) *K*-means analysis of bivalent promoters and heatmaps of H3K4me3 and H3K27me3 ChIP-seq signal intensity within ±3 kb windows around the TSS at cluster I (H3K4me3-high, with intensity of H3K4me3 higher than H3K27me3 FC ≥1.5), cluster II (H3K27me3-high, with intensity of H3K27me3 higher than H3K4me3 FC ≥1.5), and cluster III (H3K4me3/K27me3-equal, with intensity of H3K27me3 equal to that of H3K4me3 |FC|<1.5 in the indicated models. (**G**) Alluvial plot of dynamic changes of chromatin bivalency states (CBSs) in the 3 clusters from drug-sensitive cells (LNCaP) to drug-resistant cells (42D^EnzR^). (**H**) IGV snapshots of H3K27me3 and H3K4me3 ChIP-seq at a representative gene in the indicated cells and PDX models. (**I**) Scatter plot of log_2_ FC in H3K4me3 mark intensity between 42D^EnzR^ and LNCaP cells at all bivalent promoters and log_2_ FC in bivalent gene transcript levels between 42D^EnzR^ and LNCaP cells. Pearson’s correlation scores and associated *P* value are indicated.

We previously demonstrated that targeting EZH2 induced the expression of major LP genes such as *ASCL1* and *SOX2*, which displayed chromatin bivalency in an AR-negative PDX model (18). To determine whether tumor CSPs are controlled by chromatin bivalency, we first defined PCa tumor gene bivalency by performing sequential ChIP-seq, which identifies bivalent genes with high confidence (3, 34). Counting promoters that were positively detected by both sequential and separate ChIP-seq of H3K4me3 and H3K27me3, our analyses identified approximately 3,000–5,000 bona fide bivalent, protein-coding genes in fresh clinical adenocarcinoma tumors, immune-intact mouse tumors, and ARSI-resistant PDX tumors and cell models (Figure 3, A and B, Supplemental Figure 3A, and Supplemental Table 2). Notably, the bivalent genes we identified here encompassed most of the bivalent genes identified in recent studies using separate ChIP-seq or CUT&RUN (18, 35, 36). Further analysis demonstrated that bivalent genes in clinical tumors and preclinical models were highly overlapping. The overlapped genes were strongly enriched in CSPs (Figure 3, C and D, and Supplemental Figure 3, B–D). Moreover, the bivalent genes were also enriched in major regulatory programs, which include Wnt signaling, kinase signaling, response to chemical stress or insulin, and histone modification. Chromatin state analysis revealed that bivalent genes were probably regulated primarily by bivalency at their promoters (Supplemental Figure 3E).

Significantly, we found that over 70% of the PCa cell state diversity genes were bivalent. They included programs of oncogenesis (110 genes) and response to stress/hypoxia (92 genes) and IFN (86 genes), as well as lineage specification (292 genes) and EMT (173 genes) (Figure 3D). Remarkably, our integration of genes commonly upregulated by PRC2i/EHZ2i in combination with ARSIs revealed that all bivalent CSP genes were induced by this combination treatment, which accounted for over 80% of all genes that were upregulated (Figure 3E). These data clearly indicated that cell state diversity induction was closely linked to chromatin bivalency alteration. The expression of bivalent genes in the cancer cells was highly responsive to the disruption of bivalency.

In embryonic development, changes in chromatin bivalency (e.g., increase in H3K4me3 and/or decrease in H3K27me3) result in the expression of specific developmental programs (3). We thus sought to determine whether similar alterations occur in drug resistance and cell state diversity. To this end, we used a pair of ARSI drug (Enz)-sensitive (LNCaP) and Enz-resistant (42D^EnzR^) (37) cell models and a pair of Enz-sensitive (LuCaP35CR) and Enz-resistant (LuCaP35EnzR) (this study) (38) tumor models. *K*-means clustering analysis of the bivalency marks revealed that, compared with the drug-sensitive cells and tumors, the drug-resistant ones had an increased number of genes that were H3K4me3-high and a reduced number of genes that were H3K27me3-high (Figure 3F and Supplemental Figure 3F). Indeed, genes in the CSP programs switched their bivalency from H3K27me3-high in cluster II or H3K27me3-H3K4me3 equal in cluster III in drug-sensitive cells to H3K4me3-high in cluster I in drug-resistant cells (Figures 3G and Supplemental Figure 3G and Supplemental Table 3). Consistent with the role of bivalency resolution in gene induction, over 85% of genes with the bivalency switches displayed increased expression in the drug-resistant models. Among them were CSP genes such as *VEGFA*, *MYCL*, and *EPAS1* as well as cell-fate TFs (e.g., *ASCL1*, *NKX2-1* and *FOXA2*) (Figure 3H and Supplemental Figure 3, H and I). Further analysis demonstrated that bivalency changes (i.e., H3K4me3 increase or H3K27me3 decrease) at individual genes were highly correlated with changes in their mRNA expression (Figure 3I and Supplemental Figure 3J). Taken together, our results demonstrated that CSP genes were largely bivalent and that cell state diversity induction was associated with chromatin bivalency alteration.

### Cotargeting of PRC2 and the AR effectively disrupts the functional balance of PRC2 and KMT2B in bivalency maintenance.

**Figure 4:**
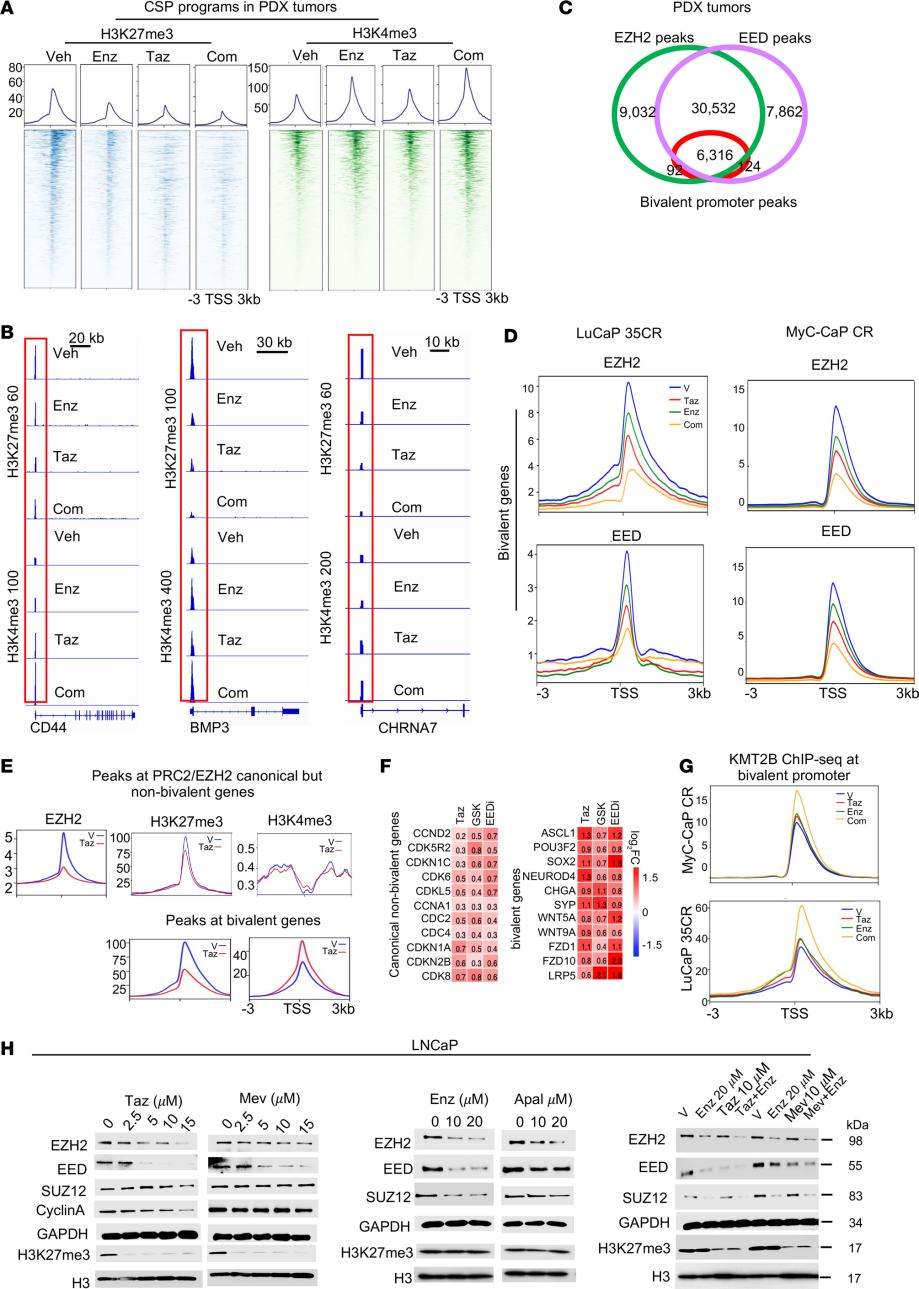
Cotargeting of PRC2 and AR effectively disrupts the functional balance of PRC2 and KMT2B in bivalency maintenance. (**A**) Heatmaps and signal profiles of H3K27me3 and H3K4me3 ChIP-seq signal intensity within ±3 kb windows at the TSS of CSPs (*n* = 798 peaks) in LuCaP35CR tumors in mice treated as indicated for 10 days. The ChIP-seq peaks were selected with FDR below 0.05. (**B**) IGV snapshots of H3K27me3 and H3K4me3 ChIP-seq peaks at representative CSP genes in LuCaP35CR tumors in mice treated as indicated for 10 days. (**C**) Venn diagram of ChIP-seq peak overlaps between EZH2 and EED at bivalent promoters in LuCaP35CR tumors. (**D**) Signal profiles of EZH2 and EED ChIP-seq within ±3 kb windows around the TSS at bivalent genes in LuCaP35CR and Myc-CaP tumors in mice treated as indicated for 10 days. The ChIP-seq peak was selected with a FDR of less than 0.05. (**E**) Signal profiles of EZH2, H3K27me3, and H3K4me3 ChIP-seq signal intensity within ±3 kb windows at the TSS at canonical but nonbivalent PRC2 target genes and at bivalent genes in 42D^EnzR^ cells treated with 10 μM Taz or vehicle for 24 hours. (**F**) Heatmaps of gene expression changes when compared with vehicle in 42D^EnzR^ cells treated as indicated for 48 hours. (**G**) Signal profiles of KMT2B ChIP-seq signal intensity within ±3 kb windows at the TSS at all bivalent gene promoters in LuCaP 35CR tumors and MyC-CaP mouse tumors treated as indicated for 10 days. (**H**) Western blots of proteins in LNCaP cells treated as indicated for 72 hours.

Consistent with the function of PRC2/EZH2 in controlling bivalency, treatment with an EZH2i (Taz) strongly decreased H3K27me3 in both PDX and immune-competent tumor models as well as in the cells. Interestingly, Taz also increased H3K4me3 in the cells and tumors (Figures 4, A and B, and Supplemental Figure 4, A and B). We also found that Enz strongly decreased H3K27me3 and increased H3K4me3 at bivalent genes of CSPs. Notably, their combined treatment resulted in even more pronounced alterations of the 2 marks.

Bivalent marks in development are “written” primarily by *KMT2B*/MLL2 and PRC2/EZH2 complexes (3). Our ChIP-seq analysis revealed that the majority of EZH2- or EED-occupied promoters were bivalent in cancer cells (64% for EZH2; 65% for EED) and tumors (70% for EZH2; 69% for EED) (Supplemental Figure 4C). Notably, promoters co-occupied by EZH2 and EED contained essentially all bivalent promoters (Figure 4C). Significantly, functional inhibition of EZH2 dramatically decreased the occupancy of EZH2 and EED at bivalent promoters in the tumors and cells. As expected, it also decreased the EZH2 occupancy and H3K27me3 at nonbivalent PRC2 targets (Figure 4, D and E). Moreover, inhibition of EED function by EED226, which disrupts the PRC2 complex, also diminished H3K27me3 and its own occupancy at the bivalent promoters (Supplemental Figure 4, A and D). Interestingly, H3K27me3 marks at bivalent promoters were more sensitive to EZH2 inhibition than those at nonbivalent PRC2/EZH2 target genes (Figure 4, E and F).

To determine whether Enz affects the function of bivalency writers, we performed ChIP-seq analysis of EZH2, EED, and KMT2B in tumors treated with Enz. Our data demonstrated that Enz alone strongly diminished the occupancy of both EZH2 and EED. Taz and Enz combination diminished their occupancy further than either one alone (Figure 4D). Interestingly, Taz and Enz treatment caused a strong increase in KMT2B occupancy at the bivalent promoters (Figure 4G). In keeping with the noncanonical function of EZH2, inhibition of EZH2 diminished its occupancy at EZH2-AR target genes without significant effects on either the H3K27me3 or H3K4me3 mark (Supplemental Figure 4E). Notably, we found that none of the bivalent promoters was occupied by the AR (Supplemental Figure 4, F and G). Intriguingly, we found that Enz at 10 μM or higher and EZH2i at 5 μM or higher were sufficient to decrease EED and EZH2 protein levels and that the Taz and Enz combination was more effective in LNCaP and C4-2B cells (Figure 4H and Supplemental Figure 4H). It is well established that chromatin occupancy of EZH2 or EED at canonical PRC2 targets is in the form of the PRC2 protein complex of EZH2, EED, and SUZ12, and that EED nucleates the PRC2 complex occupancy at chromatin by binding to the H3K27me3 mark and further activating PRC2/EZH2 catalytic activity, forming a positive feedback loop in the PRC2 function of gene silencing (1, 2, 39). The remarkable decrease in EED and EZH2 protein levels will likely disrupt the PRC2 complex occupancy at chromatin and its catalytic activity in methylation of H3K27me3. Together, our results demonstrated that inhibition of PRC2 or the AR could significantly disrupt the function of PRC2 and that their cotargeting effectively tipped the functional balance of PRC2 and KMT2B in bivalency maintenance.

### Wnt/β-catenin signaling drives changes in chromatin bivalency.

**Figure 5:**
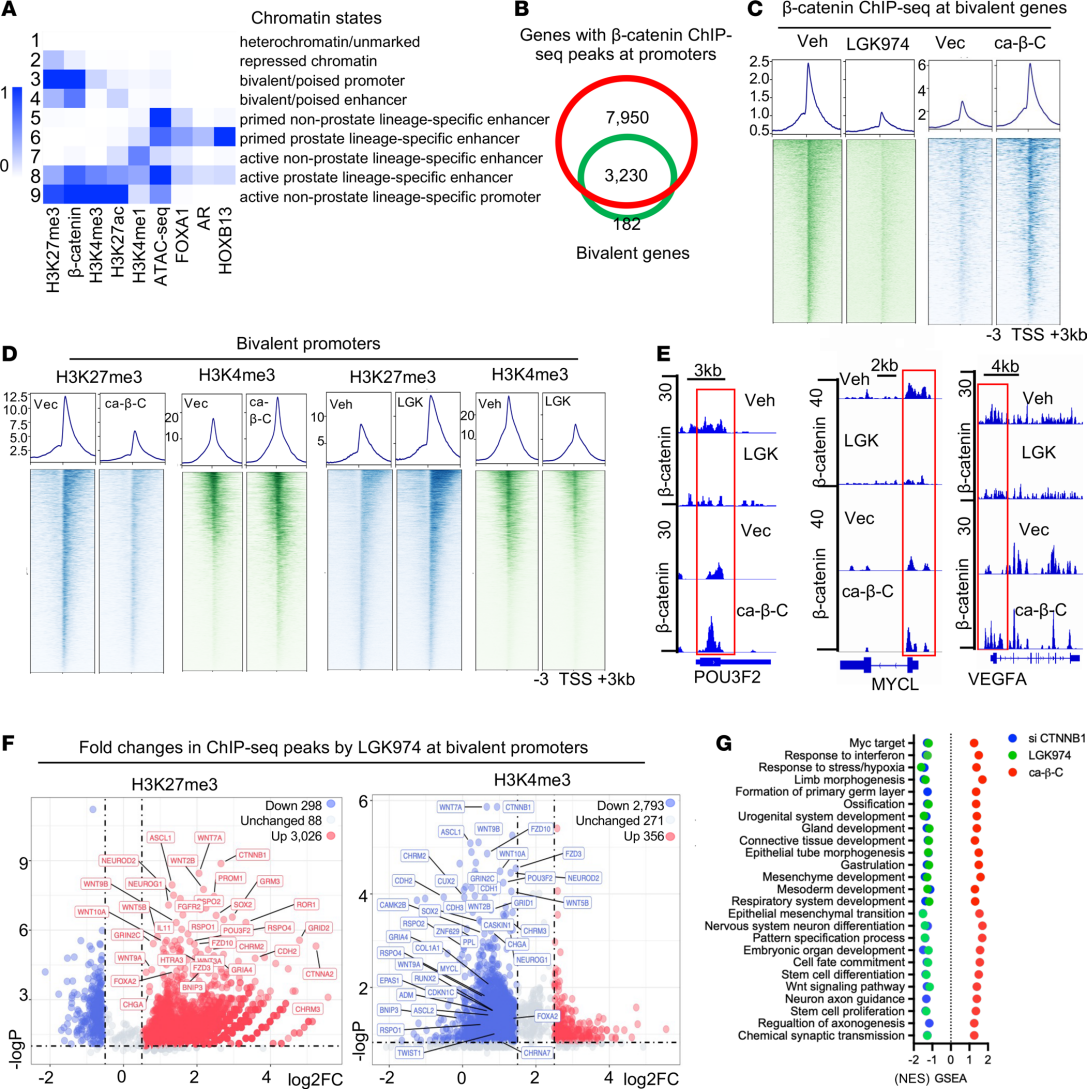
Wnt/β-catenin signaling drives chromatin bivalency changes. (**A**) Chromatin state analysis by ChromHMM of C4-2B cells with 9 epigenetic and TF ChIP-seq marks. Blue shadings depict the average intensity of a particular mark across each chromatin state. (**B**) Venn diagram of genes with β-catenin ChIP-seq peaks at promoters and bivalent genes in C4-2B cells. (**C** and **D**) Signal profiles and heatmaps of β-catenin (**C**) and H3K4me3 and H3K27me3 (**D**) ChIP-seq signal intensities within ±3 kb windows around the TSS at bivalent promoters in C4-2B cells with ca–β-C expression or vector control, or in C4-2B cells treated with 10 μM LGK974 or vehicle for 24 hours. (**E**) IGV snapshots of β-catenin ChIP-seq at the indicated bivalent genes in C4-2B cells treated as in **D**. (**F**) Volcano plots of FCs in log_2_ of H3K27me3 (left) and H3K4me3 (right) at all bivalent promoters in C4-2B cells treated with 10 μM LGK974 for 24 hours and their associated *P* values (–log_10_). (**G**) GSEA of gene expression changes detected by RNA-seq in C4-2B cells treated with 10 μM LGK974 or siβ-catenin for 48 hours, or with ca–β-C expression.

Given that in development, bivalency is mainly controlled by different signaling pathways (40), we thus examined whether any of the regulatory programs served as the potential driver for the bivalency changes. Interestingly, GO program clustering analysis of all the genes with the bivalency changes revealed that Wnt/β-catenin signaling was a potential master regulator, as it connected all the CSPs except Notch (Supplemental Figure 5A). Importantly, expression of over 70% of the Wnt signaling genes was strongly induced in the drug-resistant models, including a large number of Wnt genes (e.g., *WNT2/5A/10B*), their receptors and coreceptors (e.g., *FZD1/2/3, LRP5*), positive regulators (e.g., *DVL1, RSPO2/4*), downstream targets (e.g., *CCND3, MYC, AXIN2*), and the total and active forms of β-catenin (Supplemental Figure 5, B and C), indicating hyperactive Wnt/β-catenin signaling. To provide the direct link to chromatin bivalency, we performed β-catenin ChIP-seq followed by ChromHMM analysis and found that β-catenin chromatin occupancy was strongly associated with bivalent promoters (Figure 5A, state 3). Strikingly, close to 95% of bivalent promoters were occupied by β-catenin (Figure 5B), clearly indicating its involvement in the control of bivalency. Treatment with a Wnt signaling inhibitor (LGK974) or an ectopically expressed, constitutively active form of β-catenin (ca–β-C/CTNNB1-ΔN90) (41) strongly decreased or increased, respectively, the β-catenin occupancy exclusively at bivalent promoters including the CSPs (Figure 5, C and D, and Supplemental Figure 5, D–F).

We next investigated whether hyperactive Wnt/β-catenin signaling promotes bivalency changes in cancer progression. We first performed bivalency ChIP-seq with cells treated with ca-β-C or the Wnt inhibitor. Our results demonstrated that ca-β-C diminished H3K27me3 and increased H3K4me3. In contrast, inhibition of Wnt/β-catenin signaling caused the opposite effects. Wnt3a treatment also resulted in effects similar to those seen with ca-β-C (Figure 5E and Supplemental Figure 5, G–I). Among the 3,026 genes (of 3,412 genes) with H3K27me3 highly increased by LGK974 and 2,793 genes with H3K4me3 decreased by LGK974 were CSP genes such as *EPAS1*, *MYCL*, *BNIP3*, *ASCL1*, *NEUROD2*, *NEUROG1*, *SOX2*, and *POU3F2*. Notably, the effects were also observed for Wnt signaling genes such as *WNT7A/2B/9A/9B*, *RSPO1/2/4*, *FZD3/10*, and *CTNNB1* (Figure 5F), thus strongly suggesting a positive feed-forward loop that promotes a change in bivalency with Wnt/β-catenin signaling.

As expected, knockdown of β-catenin or Wnt inhibitor treatment markedly downregulated the expression of bivalent CSP genes, particularly in programs of MYC targets, response to stress, organ/tissue development, and EMT. In contrast, ca-β-C strongly induced their expression (Figure 5G). Indeed, the mRNA and protein expression of key LP drivers and markers such as ASCL1, BRN2, ONECUT1, CHGA, as well as of major neuron signaling and axon guidance genes was strongly affected by the treatments (Supplemental Figure 5, J and K). The expression of Wnt signaling program genes was also largely affected. Consistent with the above data, the expression of Wnt/β-catenin signaling genes was significantly correlated with the expression of other bivalent CSP genes in clinical tumors (Supplemental Figure 5L). In line with the effects on axonogenesis genes, ca-β-C significantly increased neurite-like protrusions (Supplemental Figure 5M).

### REV-ERBα mediates Wnt/β-catenin function in driving chromatin bivalency changes.

**Figure 6:**
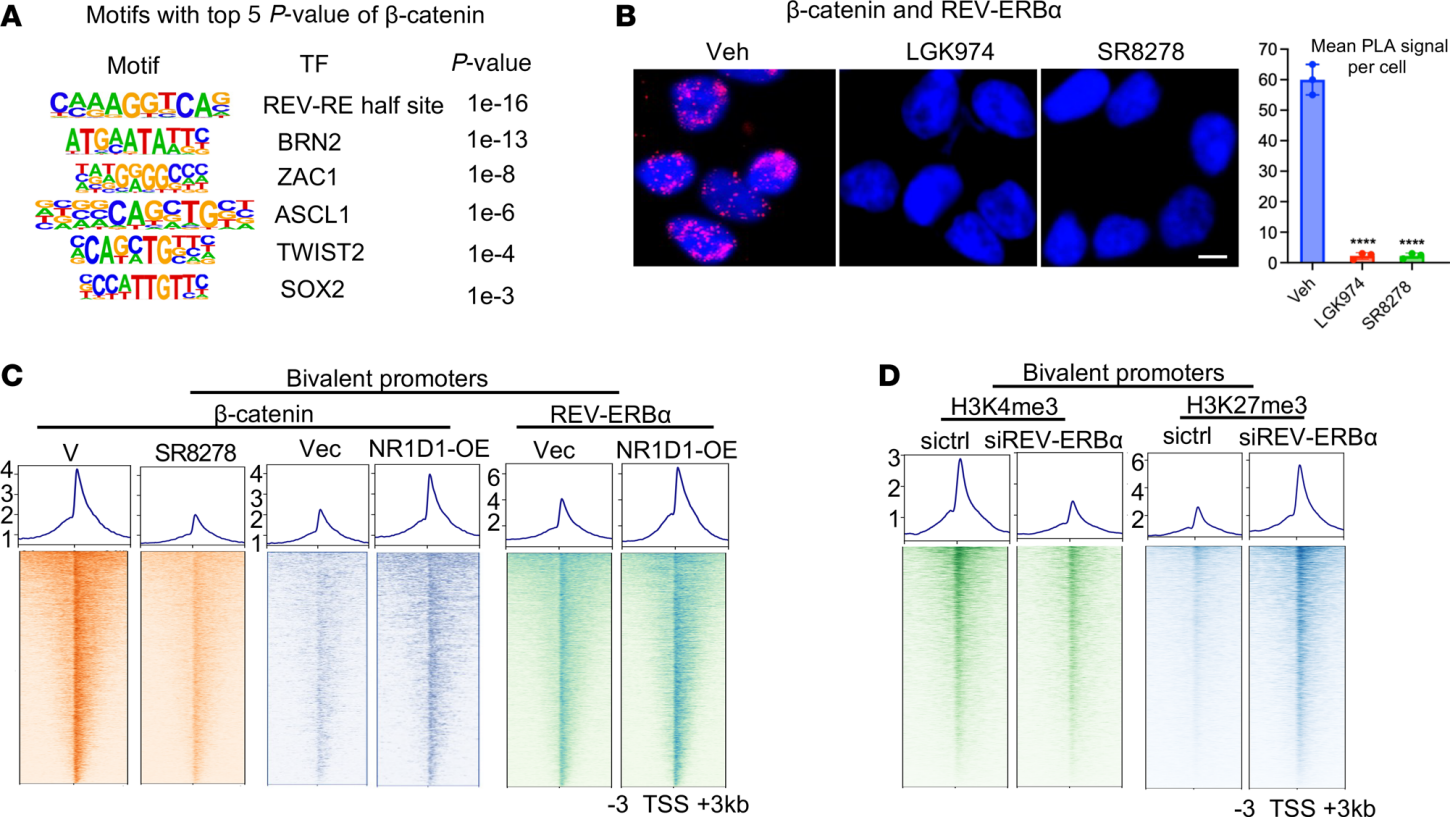
REV-ERBα mediates Wnt/β-catenin function in driving chromatin bivalency changes. (**A**) Motif analysis of β-catenin ChIP-seq peaks at bivalent promoters in 42D^EnzR^ cells. Motifs presented are based on the top 6 *P* values and distinct core sequences. (**B**) Left: Representative cell images from PLA analysis of β-catenin and REV-ERBα association in 42D^EnzR^ cells treated with 10 μM LGK974 or 7.5 μM SR8278 vehicle for 24 hours. Images are representative of 3 independent experiments. Right: PLA dots were quantified (mean ± SD; *n* = 3). Scale bar: 50 μm. ****P < 0.0001, by 2-tailed Student’s *t* test. (**C**) Signal profiles and heatmaps of β-catenin and REV-ERBα ChIP-seq peaks intensity within ±3 kb windows at bivalent promoters in 42D^EnzR^ cells treated with 7.5 μM SR8278 for 24 hours or with REV-ERBα overexpression. V, vehicle; Vec, vectors. (**D**) Signal profiles and heatmaps of H3K27me3 and H3K4me3 ChIP-seq peaks intensities within ±3 kb windows at bivalent promoters in ca-β-C–expressing C4-2B cells treated with siREV-ERBα or siControl (sictrl) for 24 hours.

To identify TF mediators of the β-catenin function, we first analyzed the potential TF binding motifs that are associated with β-catenin occupancy at bivalent promoters and found that motifs of TFs such as REV-ERBs, BRN2, ZAC1, and ASCL1, but not TCF/LEF, were highly significantly enriched (Figure 6A). We recently found that in PCa adenocarcinoma cells and tumors, REV-ERBα (encoded by NR1D1), a major circadian rhythm regulator, switches its function from repressor to an activator in stimulation of the expression of a large number of tumorigenic genes (42). Our further study revealed that REV-ERBα plays a key role in PCa LP and ARSI drug resistance (43). Thus, we examined whether REV-ERBα is a potential mediator. Indeed, we found that REV-ERBα associated with β-catenin in the nucleus, and their association was significantly disrupted by inhibitors of Wnt and REV-ERBα (Figure 6B and Supplemental Figure 6A). Our ChromHMM analysis also found that REV-ERBα chromatin occupancy is strongly associated with bivalent promoters (Supplemental Figure 6B, state 3). Close to 95% of bivalent promoters were occupied by REV-ERBα (Supplemental Figure 6C), strongly suggesting its involvement in bivalency control. Ectopic expression of REV-ERBα strongly increased the occupancy of β-catenin at bivalent genes, and REV-ERBα inhibition diminished β-catenin occupancy (Figure 6C and Supplemental Figure 6D). Knockdown of REV-ERBα in ca-β-C–expressing cells dramatically decreased H3K4me3 and increased H3K27me3 at bivalent promoters (Figure 6D). Consistent with REV-ERBα being a mediator of β-catenin function, REV-ERBα knockdown also resulted in the downregulation of genes controlled by β-catenin, including different CSPs such as the cell response to stress and IFN, the cell cycle, and Wnt signaling (Supplemental Figure 6E). Together, our results demonstrated, unexpectedly, that REV-ERBα was a mediator of Wnt/β-catenin function in driving bivalency changes.

### Hyperactive Wnt/β-catenin signaling and REV-ERBα promote KMT2B function and antagonize PRC2 in driving chromatin bivalency change.

**Figure 7:**
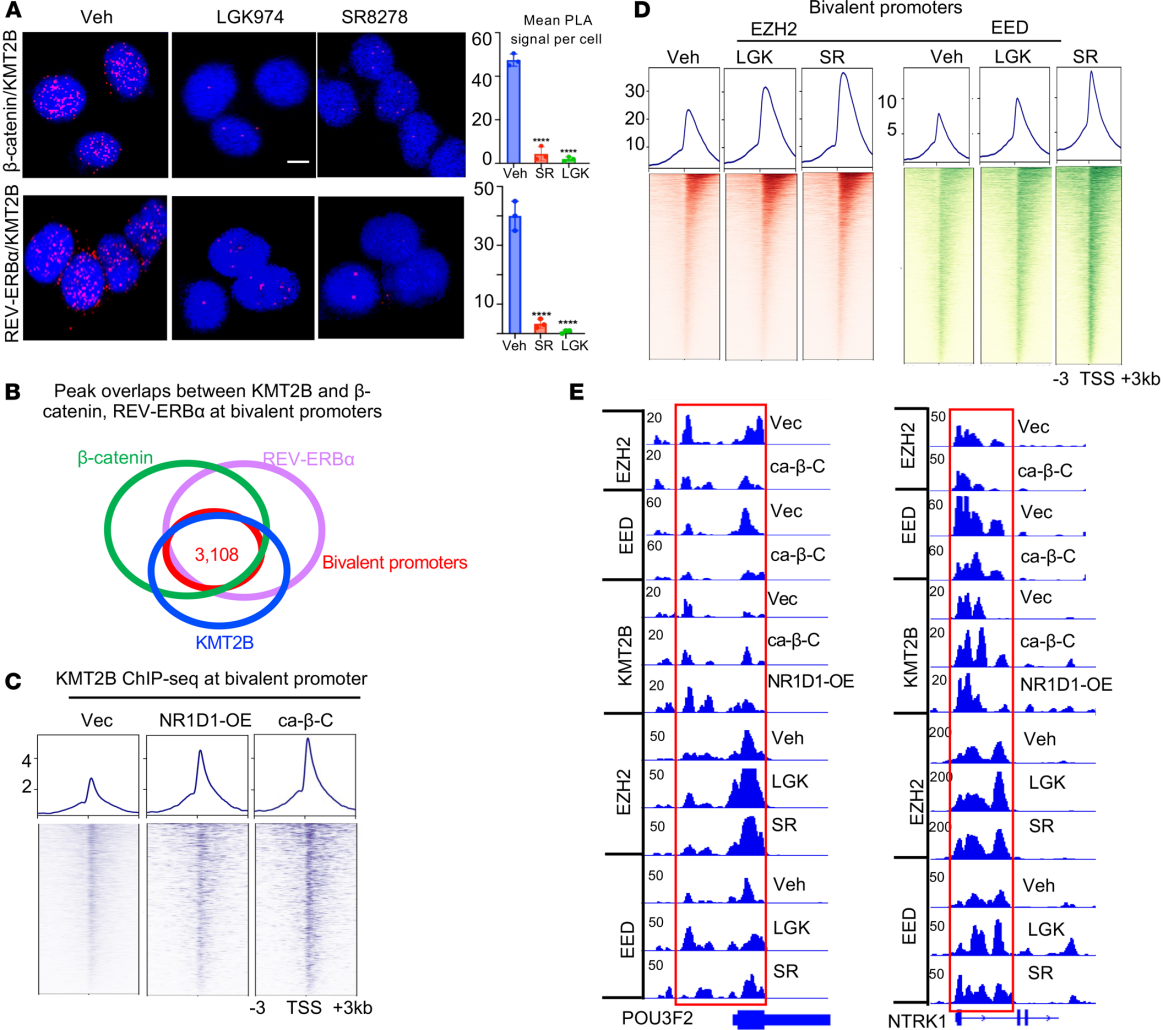
Hyperactive Wnt/β-catenin and REV-ERBα promote KMT2B function and antagonize PRC2 in driving chromatin bivalency changes. (**A**) Left: Representative images of PLA analysis of REV-ERBα, β-catenin, and KMT2B in 42D^EnzR^ cells treated with 10 μM LGK974, 7.5 μM SR8278, or vehicle for 24 hours. Images are representative of 3 independent experiments. Right: PLA dots were quantified (mean ± SD; *n* = 3). Scale bar: 50 μm. ****P < 0.0001, by 2-tailed Student’s *t* test. (**B**) Venn diagram of peaks of REV-ERBα, β-catenin, and KMT2B ChIP-seq at bivalent promoters in C4-2B cells. (**C**) Signal profiles and heatmap of KMT2B ChIP-seq within ±3 kb windows around the TSS at bivalent promoters in C4-2B cells with ca–β-C expression, REV-ERBα OE, or vector control. (**D**) Signal profiles of EZH2 and EED ChIP-seq within ±3 kb windows around the TSS at bivalent promoters in 42D^EnzR^ cells treated with vehicle,10 μM LGK974, or 7.5 μM SR8278 for 24 hours. (**E**) IGV snapshots of the indicated ChIP-seq at the indicated bivalent genes in C4-2B cells treated as in **C** and **D**.

To examine whether β-catenin and REV-ERBα promotes bivalency changes through the bivalent writer complexes, we first performed a proximity ligation assay (PLA). Indeed, we detected abundant interactions between KMT2B and β-catenin and REV-ERBα in nuclei, which were largely disrupted by the inhibitors of Wnt and REV-ERBα (Figure 7A). We then performed KMT2B ChIP-seq and found that, like β-catenin and REV-ERBα, a major proportion of KMT2B chromatin occupancy was at bivalent genes (Figure 7B). Ectopic expression of ca-β-C and REV-ERBα significantly increased KMT2B distribution at proximal promoter regions genome wide (Supplemental Figure 7A) and enhanced KMT2B occupancies at bivalent promoters (Figure 7C). ChIP-re-ChIP demonstrated co-occupation of β-catenin and REV-ERBα with KMT2B at the bivalent promoters and that their co-occupancy was significantly decreased by the inhibitors of Wnt and REV-ERBα. Furthermore, knockdown of β-catenin and REV-ERBα strongly decreased the occupancy of KMT2B and H3K4me3 at the bivalent promoters (Supplemental Figure 7, B and C).

We then sought to determine whether Wnt/β-catenin and REV-ERBα also affect the function of EZH2/PRC2. Indeed, inhibitors of Wnt signaling and REV-ERBα markedly increased the occupancy of EZH2 and EED at bivalent promoters (Figure 7, D and E) without apparent effects on nonbivalent promoters (Supplemental Figure 7D). Knockdown of β-catenin and REV-ERBα also increased the occupancy of EZH2 and H3K27me3 mark at bivalent promoters (Supplemental Figure 7E). Together, these findings suggest that β-catenin and REV-ERBα promote bivalency changes by recruiting KMT2B and antagonizing PRC2/EZH2 occupancy.

### Induction of tumor cell–state diversity by PRC2/EZH2 inhibition can be mitigated by Wnt/β-catenin signaling blockade through alteration of chromatin bivalency.

**Figure 8:**
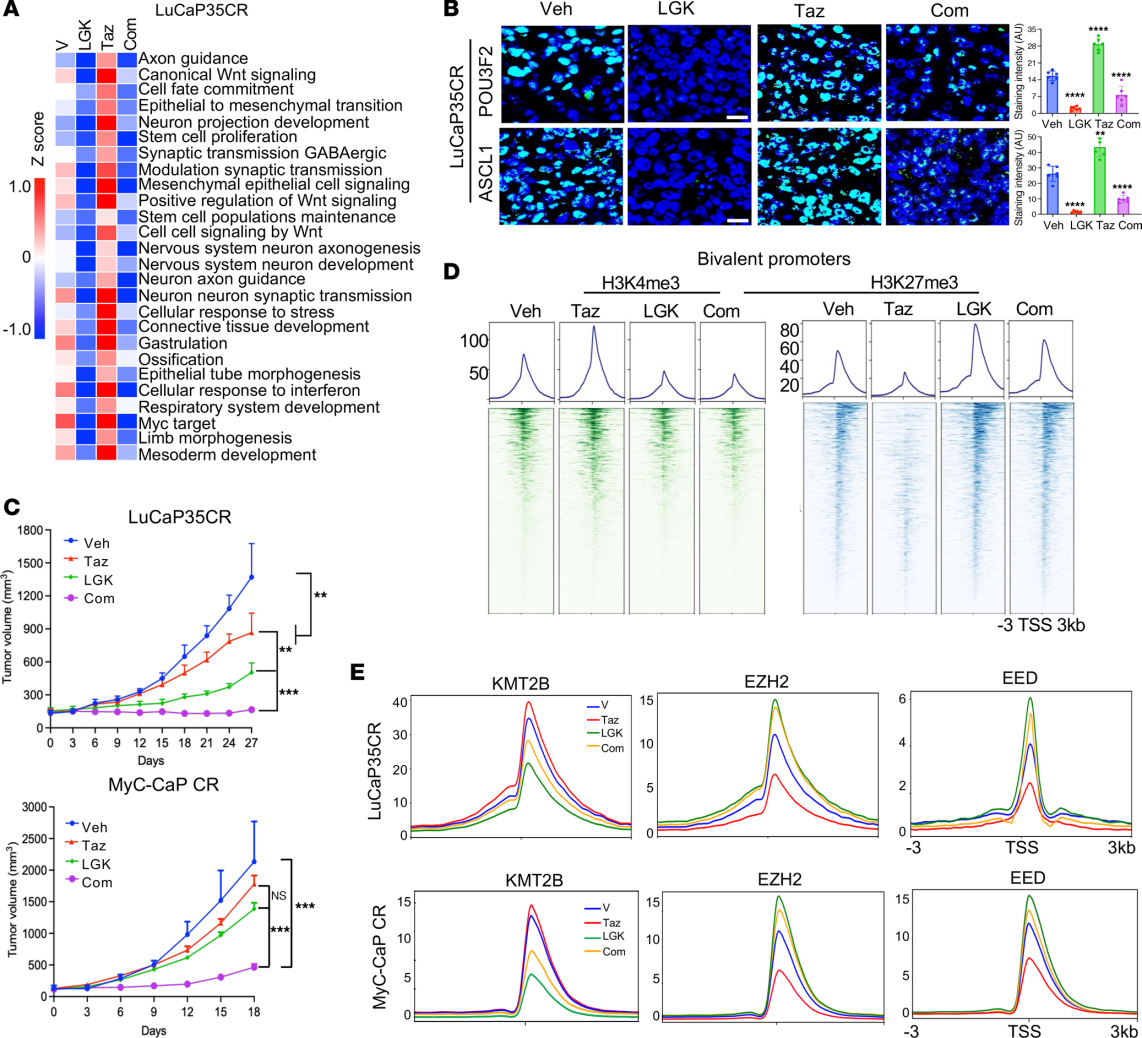
Induction of tumor cell–state diversity by PRC2/EZH2 inhibition can be mitigated by Wnt/β-catenin signaling blockade through alteration of chromatin bivalency. (**A**) Heatmaps of the GSVA score of the indicated programs detected by RNA-seq in LuCaP35CR tumors in mice treated as indicated for 10 days. (**B**) Representative confocal microscopy images of LP protein expression in LuCaP35CR tumors in mice treated as indicated (*n* = 5 mice per group). Note: The experiment here shares samples of vehicle and Taz treatments with those in Figure 2C and Supplemental Figure 2H. Scale bars: 50 μm. Bar graphs display ImageJ-analyzed IF intensity from 7 randomly selected fields per tumor. ****P < 0.0001, by 2-tailed Student’s *t* test. (**C**) Growth curves of LuCaP35CR PDX and MyC-CaP CR tumors in mice treated with vehicle or the indicated compounds for the indicated durations, 7 times per week (*n* = 5 mice per group). **P < 0.01 and ***P < 0.001, by 2-tailed Student’s *t* test. (**D**) Signal profiles and heatmaps of the indicated histone marks at bivalent promoters in LuCaP35CR tumors in mice treated as indicated for 10 days. (**E**) Signal profiles of EZH2, EED, and KMT2B/MLL2 ChIP-seq within ±3 kb windows around the TSS at bivalent CSPs in LuCaP35CR and MyC-CaP CR tumors in mice treated as indicated for 10 days.

Given that hyperactive Wnt/β-catenin signaling antagonizes the function of PRC2, we examined whether therapeutic targeting of Wnt/β-catenin is sufficient to mitigate the unwanted consequences of PRC2/EZH2 inhibition. Indeed, addition of the Wnt inhibitor LGK to Taz treatment effectively eliminated the induction of all CSPs by Taz in both PDX and immune-competent tumors (Figure 8, A and B, and Supplemental Figure 8, A–C). It also significantly decreased the protein expression of ASCL1, POU3F2, and SOX2 and the marker CD44 in the tumors. Interestingly, LGK974 addition also decreased the expression of genes controlling cell proliferation and survival more effectively than either one alone (Supplemental Figure 8D). Consistently, the combination of LGK and Taz was more effective at inhibiting tumor growth than either one alone in both PDX and immune-competent tumors (Figure 8C and Supplemental Figure 8, E and F).

Consistent with the changes in gene expression, LGK addition was effective in restoring the H3K27me3 mark at bivalent promoters that were diminished by Taz and in abolishing H3K4me3 increases by Taz, thus tipping the bivalency dynamics toward silencing (Figure 8D and Supplemental Figure 8G). In line with the changes in bivalency, LGK addition effectively countered Taz-induced reduction in EZH2 and EED occupancies, pushing their levels even above those seen with the vehicle control, while exerting a potent suppression on KMT2B occupancy (Figure 8E and Supplemental Figure 8H). In conclusion, our results demonstrated that induction of distinct tumor cell states, including LP, by PRC2/EZH2 inhibition can be effectively mitigated by blocking Wnt/β-catenin signaling through alteration of chromatin bivalency.

## Discussion

Cancer cell–state diversity involves selective co-option of partial developmental and homeostatic programs (11, 44). Through chromatin bivalency profiling, we found that the majority of CSP genes were bivalent. Importantly, disruption of chromatin bivalency induced the expression of CSPs and a spectrum of cell states marked by coexpression of genes in different developmental programs. Therefore, our findings suggest that bivalency is an important constraint of cell state diversity.

Wnt/β-catenin signaling plays a pivotal role in embryonic cell-fate specification and progenitor cell pluripotency as well as in cancer progression (45, 46). However, whether it directly controls chromatin bivalency has been unclear. The present study revealed that in tumors, hyperactive Wnt/β-catenin signaling drove bivalency changes to allow the induction of CSPs by promoting KMT2B function in H3K4me3 marking and by antagonizing PRC2/EZH2 in H3K27me3 deposition. Intriguingly, β-catenin did not act through TCF/LEF but rather through REV-ERBα. Such a partnership between the circadian regulator and β-catenin in coordinated regulation of bivalency was unanticipated. It is well established that in circadian rhythm, REV-ERBα acts as a repressor in its contribution to gene expression undulation. We recently found that in PCa and other cancers, REV-ERBα switches its function from a repressor to an activator through association with coactivators such as BRD4 and p300 in the expression of multiple kinase signaling pathway programs that are mostly nonbivalent (42). Remarkably, in PCa progression, ARSIs reprogram the function of REV-ERBα by switching its target gene programs to control LP (43). Data in this study revealed that one major mechanism of REV-ERBα functions is its recruitment of β-catenin to promote chromatin bivalency resolution and, consequently, induction of CSPs. Therefore, these findings not only highlight the diverse functions of REV-ERBα but also indicate an unexpected connection between loss of the circadian rhythm and induction of CSPs in cancer.

Another intriguing finding here is that most of the Wnt signaling genes were bivalent and subject to their own activation, therefore constituting a positive feed-forward loop in driving bivalency resolution. In advanced cancer such as metastatic castration-resistant prostate cancer (mCRPC), frequent genetic or nongenetic alterations of multiple components of Wnt pathways result in hyperactive signaling in an autocrine and/or paracrine fashion, which in turn promotes tumor cell dedifferentiation into CSCs and tumor heterogeneity. In mCRPC, therapies with potent AR antagonists compromise both canonical and noncanonical functions of EZH2. Although the reduction of noncanonical EZH2 function is likely beneficial to tumor growth inhibition, diminished canonical function of EZH2, particularly in tumors with hyperactive Wnt signaling, would shift the bivalency balance toward H3K4me3-high and/or H3K27me3-low status and result in low-level induction of CSP genes. Under such conditions, adding PRC2i will likely accelerate bivalency resolution and lead to full-fledged CSP induction as observed here.

PRC2/EZH2 function in cancer is multifaceted and often context dependent. Recent preclinical studies showed that in certain solid cancer subtypes or conditions, cotargeting PRC2/EZH2 and other specific pathways can achieve excellent antitumor effects (9). However, adverse effects and even treatment-related secondary malignancies in the clinic have recently been reported (47, 48). In PCa, the function of PRC2/EZH2 as an AR coactivator has provided a rationale of clinical trials with drugs targeting EZH2 and the AR for patients with advanced disease. We show here in multiple preclinical models that inhibitors of PRC2/EZH2 in combination with an ARSI drug effectively disrupted the function of PRC2/EZH2 in controlling the tumor CSP, enhanced tumor cell drug resistance, and promoted tumor metastasis. Although, ARSIs such as Enz have been shown to promote metastasis (13), inhibitors of PRC2/EZH2 have not been reported to display such activity. Remarkably, we revealed here that PRC2i/EZH2i alone induced invasion and that their combination with ARSIs resulted in more than additive induction of invasion. Indeed, the combination treatment induced liver metastasis.

Mechanistically, we showed that dual inhibition was closely linked to cell state reprogramming. We found that the treatments significantly increased nonluminal cell states including the neural-stem hybrid cell state, EMT, and activation of developmental and stress-response programs. Expansion of cell populations with such CSPs will likely diminish the overall reliance of the tumor on AR signaling and PRC2-regulated luminal transcriptional programs, thus resulting in reduced sensitivity to both AR antagonism and EZH2 inhibition. These findings raise a cautionary note for the treatment of advanced diseases with drugs targeting PRC2/EZH2. Importantly, we also found that the effects of PRC2/EZH2 inhibition on bivalency and cell states could be effectively mitigated by blocking Wnt/β-catenin signaling, suggesting that cotargeting PRC2/EZH2 and Wnt/β-catenin can be a valuable therapeutic strategy for more effective treatment of certain advanced cancers.

## Methods

### Sex as a biological variable.

Our study examined male animals and male individuals, as PCa only affects males.

### Cell culture.

LNCaP, 22RV1, C4-2B, and 42D^EnzR^ cells were cultured in RPMI1640 supplemented with 10% FBS. Myc-CaP cells were cultured in DMEM supplemented with 5% FBS. Cells were grown at 37°C in 5% CO_2_ incubators. Cells were obtained from the American Type Culture Collection (ATCC), except as indicated below. C4-2B was from UroCor Inc. 42D^EnzR^ was a gift from Amina Zoubeidi (Vancouver Prostate Centre, Vancouver, Canada). The cancer cell lines were recently authenticated by ATCC using short tandem repeat (STR) profiling. Cell lines were regularly tested for mycoplasma negativity.

### PDX models.

CRPC LuCaP35CR and LuCaP77CR were imported from Eva Corey’s laboratory at the University of Washington (Seattle, Washington, USA). Establishment of Enz-resistant LuCaP PDX models was recently described (49). Briefly, male CB-17/IcrHsd-Prkdc SCID mice (aged 6–8 weeks; Envigo) were castrated and 10 days later implanted s.c. with LuCaP35CR and LuCaP77CR tumor fragments approximately 4 mm^3^in size. When the tumors reached 100 mm^3^, the mice were treated with Enz (MedChem Express, TX; 20 mg/kg in 15% Cremophor, 80% PBS and 5% DMSO) by oral gavage 5 times per week for 3 weeks (LuCaP77CR) or 6 weeks (LuCaP35CR) before the tumors grew back to approximately 600–1,000 mm^3^. The tumors were then dissected and reimplanted into additional castrated mice, which received a similar Enz treatment before the tumors grew back again and were then considered Enz-resistant tumors.

### Myc-CaP syngeneic tumor model.

WT FVB/NJ mice (aged 6–8 weeks; Envigo) were injected s.c. with 5 × 10^5^ of Myc-CaP cells/mouse. When tumors reached 500 mm³, tumor-bearing mice were surgically castrated to produce castration-resistant (CR-resistant) tumors. When tumors regrew to 500 mm³ in size, they were passaged through 3 rounds of surgically castrated FVB/NJ mice before they were used as CR tumors.

### PDX tumor and syngeneic tumor treatments.

For the 10-day treatment, when LuCaP35CR and Myc-CaP CR tumors reached approximately 200 mm^3^ in size, they were randomized into 6 groups (*n* = 5–7 per group) for daily treatments as follows: (a) vehicle for Wnt/β-catenin inhibitor or EZH2 antagonist (100 μL 5% DMSO and 95% safflower oil, p.o.); (b) EZH2 antagonist Taz/EPZ6438 (50 mg/kg in 5% DMSO, 95% safflower oil, p.o.); (c) Wnt/β-catenin inhibitor LGK974 (10 mg/kg in 5% DMSO, 95% safflower oil, p.o.); (d) combination of 50 mg/kg Taz and 10 mg/kg LGK974 (5% DMSO, 95% safflower oil, p.o.); (e) Enz (20 mg/kg in 5% DMSO, 95% safflower oil, p.o.); (f) combination of 50 mg/kg Taz and 20 mg/kg Enz (5% DMSO, 95% safflower oil, p.o.). Animals were sacrificed 10 days later with tumors harvested for OCT embedding, ChIP-seq, RNA-seq, and/or scRNA-seq analyses.

For assessment of the inhibitors’ effects on tumor growth, LuCaP35CR tumors and Myc-CaP-CR tumors were randomized to 4 groups (*n* = 5–7) per group as follows: (a) vehicle for Wnt/β-catenin inhibitor or EZH2 antagonist (100 μL 5% DMSO and 95% safflower oil, p.o.); (b) EZH2 antagonist Taz (50 mg/kg in 100 μL 5% DMSO and 95% safflower oil, p.o.); (c) Wnt/β-catenin inhibitor LGK974 (10 mg/kg in 100 μL 5% DMSO and 95% Safflower oil p.o.); (d) combination of 50 mg/kg Taz and 10 mg/kg LGK974. Mice were treated with indicated compounds daily and tumors were measured using calipers every 3 days. Tumor volumes were calculated using (length × width^2^)/2. Mice were sacrificed and tumors were harvested when vehicle-treated tumors reached approximately 1,000 mm^3^.

### Orthotopic tumor implantation and in vivo bioluminescence imaging.

For intraprostatic implantation, Myc-CaP-Luc cells were implanted. They were derived by infection of Myc-CaP cells with lentiviruses that express luciferase which were generated with pLX307-luciferase plasmid (Addgene, 117734). Prior to orthotopic implantation, Myc-CaP-Luc cells were counted, resuspended in 1:1 PBS/Matrigel solutions at a final concentration of 1 × 10^6^ cells/30 μL and kept on ice until injection to prevent solidification. Six- to 8-week-old FVB/NJ mice were anesthetized with isoflurane inhalation and a low abdominal transverse incision was made through the skin and abdominal muscle to expose the prostate. The cell suspension (30 μL) was injected into the anterior lobe of prostate and waited for approximately 30 seconds to allow Matrigel to partially solidify within the lobe. The bladder and prostate were returned to the abdomen, and the incision was closed using sutures. For bioluminescence tumor imaging, mice were i.p. injected with 10 μL/g body weight of luciferin (Gold Biotechnology, NC0107335) and imaged with a LagoX Spectrum Imaging System. Images were analyzed using Aura Living Image Software and significance were statistically analyzed using GraphPad software.

### ChIP-seq, sequential ChIP-seq and ChIP-qPCR.

ChIP-seq was performed following procedures described previously (42). Briefly, for β-catenin, KMT2B, EZH2 and EED ChIP-seq, approximately 2 × 10^7^ cells were subjected to crosslinking in 1% formaldehyde for 8 minutes followed by quenching with glycine for 5 minutes on ice. For histone mark ChIP-seq, approximately 1 × 10^7^ cells were used. Processing tumor tissues for ChIP has been described recently (42). For sequential ChIP, the first antibody-immunoprecipitated complexes (e.g., anti-H3K27me3) were washed and eluted in 50 mM NaHCO_3_, 1% SDS, 10 mM DTT, followed by overnight reverse crosslinking. The eluted ChIP complex was diluted with ChIP IP buffer and incubated with the second antibody (e.g., normal IgG or anti-H3K4me3) overnight at 4°C, before being processed for precipitated genomic DNA isolation. Libraries were sequenced in paired-end 50 bp mode on the Illumina HiSeq 2000 Sequencer (Novogene). Antibodies used for the ChIP-seq are REV-ERBα (Proteintech, 14506-1-AP, 5 μg; and Cell Signaling Technology, catalog 13418, 5 μg), H3K27me3 (MilliporeSigma, catalog 07-449, 5 μg; Cell Signaling Technology, catalog 9733, 5 μg), H3K4me3 (MilliporeSigma, catalog 07-473, 5 μg; Cell Signaling Technology, catalog 9727, 5 μg), β-catenin (Cell Signaling Technology, catalog 8480, 5 μg; Thermo Fisher Scientific, catalog 71-2700, 5 μg), EZH2 (Diagenode, catalog C15410039-50, 5 μg; Cell Signaling Technology, catalog 5246S, 5 μg), EED (Active Motif, catalog 61203, 5 μg; Cell Signaling Technology, catalog 85322, 5 μg) and KMT2B (Cell Signaling Technology, catalog 63735S, 5 μg).

### ChIP-seq data analysis.

Fastq files from ChIP-seq or sequential ChIP-seq were processed by the pipeline of ENCODE Transcription Factor and Histone (https://github.com/ENCODE-DCC/chip-seq-pipeline2). Briefly, sequencing tags were mapped against the *Homo sapiens* (human) reference genome (hg19) and *Mus musculus* (mouse) reference genome (mm10) using BWA 0.7.15. Uniquely mapped tags after filtering and de-duping were used for peak calling by model-based analysis for ChIP-seq (MACS 2.1.0) to identify regions of enrichment over background. Normalized genome-wide signal coverage tracks from raw-read alignment files were built by MACS2, UCSC tools (bedGraphToBigWig/bedClip; http://hgdownload.cse.ucsc.edu/admin/exe/linux.x86_64/) and bedTools (https://github.com/arq5x/bedtools2). Visualization of ChIP-seq signal at enriched genomic regions (avg profile and heatmap) was achieved by using deepTools (https://deeptools.readthedocs.io/en/develop/index.html). Peak associated genes were identified using the annotatePeaks function of HOMER with default settings (http://homer.ucsd.edu/homer/index.html). Each peak was assigned to the nearest gene. Further annotation information includes whether a peak is in the transcription start site (TSS) (from –1 kb to +100 bp), the transcription termination site (TTS) (from −100 bp to +1 kb), exon (coding), 5′-UTR, 3′-UTR, intronic, or intergenic. Motifs enriched within β-catenin–binding sites were identified using Homer findMotifGenome.pl with argument “hg19-p32” to detect enrichment of de novo and known TF motif. Those motifs with high background enrichment and possible false-positives were removed. The region size was 200 bp.

### Chromatin states analysis.

We used the chromatin state segmentation software ChromHMM (version 1.14) to compute genome-wide chromatin state predictions in each condition based on relative enrichment levels of histone modifications, TFs, DNA methylation and chromatin accessibility. Five epigenetic marks (H3K27ac, H3K27me3, H4K20me3, H3K4me1, and H3K4me3), four TFs (AR, FOXA1, HOXB13 and β-catenin), chromatin accessibility (ATAC-seq) (GSE260727) and DNA methylation (GSE158927) were used to construct the ChromHMM model. We used default parameters of 200 bp to partition the genome into ChromHMM categories. The state model was generated based on the levels of enrichment of each histone modification, TFs, DNA methylation and chromatin accessibility. Conservation of each state was determined using phastCons conservation scores and comparing across samples via Wilcoxon rank-sum and Kruskal-Wallis tests.

### Bivalent gene identification and analysis.

We identified bivalent genes at promoter regions of TSS ± 1 kb, using data from sequential ChIP-seq (H3K27me3→H3K4me3) and H3K27me3 and H3K4me3 separate ChIP-seq. The overlapped genes from both ChIP-seq and sequential ChIP-seq experiment are defined as bona fide bivalent genes. We performed functional analysis of the bivalent genes using GO in R (version 4.1.0). The most enriched pathways of bivalent genes were identified and selected according to the *P* value in log_10_. For bivalency alteration analysis, we compared the ChIP-seq peak height at promoters between treatment and control samples using Manorm 2.0 software with a statistical cutoff (*P* < 0.05). We selected the bivalent genes with H3K27me3 or H3K4me3 peak changed with a log_2_ fold change (FC) of –0.58 or less or a log_2_ FC of 0.58 or greater.

### Bulk RNA-seq and analysis.

For RNA isolation from cells, LNCaP, C4-2B and 42D^EnzR^ cells were treated with siRNA-β-catenin or siRNA-Control for 48 hours, or with vehicle, 10 μM LGK974, 10 μM Taz, 10 μM GSK126 and 10 μM EED226 for 48 hours. For RNA isolation from tumors, mice with LuCaP35CR tumors and Myc-CaP-CR tumors were treated with 20 mg/kg Enz, 50 mg/kg Taz, 10 mg/kg LGK974, Enz and Taz combination and Taz and LGK combination for 10 days, before tumors were dissected for RNA extraction. RNA-seq libraries from 1 μg total RNA were prepared using Illumina TruSeq RNA Sample Prep Kit, according to the manufacturer’s instructions. Libraries were validated with an Agilent Bioanalyzer (Agilent Technologies). The sequencing was performed on an Illumina HiSeq 2000 sequencer at Novogene. The fastq-formatted sequence data were analyzed using a standard BWA-Bowtie-Cufflinks workflow. Sequence reads were mapped to GRCh37/hg19 or GRCm38/mm10 assembly with BWA and Biotie software. The Cufflinks package was used for transcripts assembly, quantification of normalized gene and isoform expression, and analysis of different expression.

### scRNA-seq and data analysis.

For scRNA-seq with PDX tumors, homogenized tissues were passed through 40 μm filters and were subject to removal of RBCs, before being resuspended in cold PBS and subject to scRNA-seq procedures of 10X genomics. We analyzed scRNA-seq data using Cell Ranger version 3.2. The gene expression matrix was processed and analyzed by Seurat (version 5.0) and an R toolkit (https://github.com/satijalab/seurat), using the software R (version 4.2.0). We performed Seurat-based filtering of cells based on the number of detected genes per cell (200–5000) and the percentage of mitochondrial genes expressed (<10%). Counts were then normalized (Seurat:NormalizeData, method = logNormalize, scale.factor = 10000) and the top 3000 most variable features were selected (Seurat:FindVariableFeatures, method = vst). Data were then scaled (Seurat:ScaleData) and PCA was performed up to the top 30 components (Seurat:RunPCA). We proceeded to the determination of the *k*-nearest neighbors of each cell and the construction of a shared nearest-neighbor (SNN) graph (Seurat:FindNeighbors). We then identified clusters using the SNN modularity optimization-based clustering algorithm (Seurat:FindClusters, resolutio*n* = 0.8). Finally, we performed UMAP dimensionality reduction on the first 30 PCs, annotated the previously identified clusters, and generated plots accordingly. Clusters were associated with cell types on the basis of the scores of differential expression of well-established marker genes for each cell type: endothelia cells (*CLDN5*, *VWF*, *ENG*), myofibroblasts (*RGS5*, *GJ4*, *MYH11*), luminal cells (*KRT8*, *KRT18*, *AR*), mesenchymal cells (*ZEB1*, *ZEB2*, *SANI1*, *TWIST1*, *VIM*, *CDH11*), mesenchymal-stem hybrid cells (*ZEB1*, *ZEB2*, *TWIST1*, *VIM*, *CDH11*, *CD44*, *PROM1*, *NANOG*, *KIT*, *NES*, *KLF4, CD55*, *ALCAM*, *NOTCH4*, *SOX2*, *POU5F1*), and neural-stem hybrid cells (*ALCAM*, *CD44*, *KLF4*, *SOX2*, *ASCL1*, *FOXA2*, *NKX2-1*, *MYCN*, *POU3F2*, *NSM1*, *SIAH2*, *CAM1*, *CHGA*, *CHGB*, *SYP*, *ENO2*).

### Identification of gene MPs in scRNA-seq data.

As previously described (12, 21), we used NMF analysis to identify MPs of clinical scRNA-seq datasets (GSE137829, PRJNA699369, E-MTAB-9930, GSE141445, and GSE176031) and our scRNA-seq data, focusing on epithelial cells with different treatments. NMF was performed using different values (*k* = 4, 5, 6, 7, 8 and 9), generating different programs for each tumor. We reasoned that the most meaningful NMF programs are those that would recur across different values of *k* as well as across tumors. The NMF programs were then clustered according to Jaccard similarity and were defined using the top 90 genes with the highest NMF scores. Finally, we identified 35 MPs and assessed their enrichment in functionally annotated gene sets.

### PLA.

A PLA was performed as described previously (42). Briefly, C4-2B cells (1 × 10^5^ cells) were plated in an 8-slide plate and treated with 7.5 μM LGK974 or vehicle for 24 hours. The cells were fixed with 4% PFA, washed with PBS, and treated with 0.01% Triton-100 to permeabilize the cells. After washing and blocking the cells, primary antibodies against β-catenin (BD Biosciences, catalog 610153), BRN2/POU3F2 (Cell Signaling Technology, catalog 12137), KMT2B (Cell Signaling Technology, catalog 63735S) — all diluted at 1:1,000 — and Rev-Erbα (Cell Signaling Technology, catalog 13418, diluted 1:500) were added to stain the cells overnight at 4°C, and secondary antibodies were incubated for 1 hour at 37°C. After ligation and amplification, the slides were mounted for imaging in a confocal microscope (Zeiss).

### IF analysis.

LuCaP35CR tumor cryosections were fixed with ice-cold paraformaldehyde in 4% PBS (Thermo Fisher Scientific) for 15 minutes, and permeabilized by 0.1% Triton X-100 for 10 minutes at room temperature. After blocked with blocking buffer (10% FBS, 0.1% Triton X-100 in PBS) for 1 hour at room temperature, the cryosections were incubated with primary antibodies against ASCL1 (Abcam, catalog ab211327), BRN2 (Cell Signaling Technology, catalog 12137), CD44 (BD Pharmingen, catalog 555478), SOX2 (Abcam, catalog ab93689) at 1:100 dilution in blocking buffer at 4°C overnight. Thereafter, sections were incubated with Alexa Fluor 488–conjugated anti-mouse (Invitrogen, Thermo Fisher Scientific, catalog A11001) antibodies, Alexa Fluor 488–conjugated anti-rabbit (Invitrogen, Thermo Fisher Scientific, catalog A11008), Alex Fluor 555–conjugated anti-rabbit (Invitrogen, catalog A21429) antibodies or Alex Fluor 594–conjugated anti-rabbit (Invitrogen, Thermo Fisher Scientific, catalog A11037) antibodies at 1:200 dilution in blocking buffer for 1 hour at room temperature. Then washed slides were incubated with 1 μg/mL Hoechst (Invitrogen, Thermo Fisher Scientific, catalog H1399) for 5 minutes at room temperature to visualize nuclei. The stained sections were visualized with a Zeiss LSM 800 Airyscan Microscope using Zeiss ZEN. IF staining intensity was quantified by ImageJ software (NIH).

### Cell migration and invasion.

Cell migration and invasion assays were performed using Transwell chambers (8.0 μm pore size, Corning) in 12-well plates. For migration assays, the upper chambers were left uncoated. For invasion assays, the upper surface of the membrane was coated with 50 μL Matrigel (Corning, diluted 1:4 in serum-free medium) and allowed to polymerize at 37°C overnight. A total of 1 × 10^5^ cells were seeded in the upper chamber in FBS-free medium containing 10 μM Taz, 10 μM Enz, or their combination, and medium containing 20% FBS was added to the lower chamber. Plates were incubated at 37°C in 5% CO_2_ for 24 hours for migration and 48 hours for invasion. Nonmigrated/noninvaded cells were removed from the upper surface of the membrane using a cotton swab. Cells on the lower surface were fixed in 70% ethanol for 10 minutes and stained with 1% crystal violet for 10 minutes. Stained cells were imaged using a bright-field microscope, and 5 random fields per insert were quantified using ImageJ.

### Cell growth and IC_50_ determination.

Cells were treated with either Enz, Taz, Mev, or EED226 alone or a combination of Enz with Taz, Mev, or EED226 for 10 days. Then cells were seeded in 96-well plates at 1,500–2,500 cells per well, corresponding to specific cell lines in a total volume of 100 μL media, and then treated with the indicated drugs, which included cabozantinib, capivasertib, alisertib, niraparib, rucaparib, cabazitaxel, and docetaxel. After 4 days of incubation, Cell-Titer Glo reagents (Promega) were added, and luminescence was measured on a GLOMAX microplate luminometer (Promega), according to the manufacturer’s instructions. All treatments were set up as triplicates, and the entire experiments were repeated 3 times. The estimated IC_50_ values were calculated using GraphPad Prism 8 (GraphPad Software). For cell growth, cells were seeded in 6-well plates at 2 × 10^5^ cells per well and treated as indicated. Total viable cell numbers were counted with the Countess II (Invitrogen, Thermo Fisher Scientific).

### Chemicals.

Taz/EPZ6438 (S7128), Mev/PF06821497 (S9655), EEDi/EED226 (S8496), and capivasertib (S8019) were from Selleck Chemicals. Enz (HY-70002), cabozantinib (HY-13016), LGK974 (HY-17545), alisertib (HY-10971), niraparib (HY-10619), rucaparib (HY-10617A), cabazitaxel (HY-15459), and docetaxel (HY-B0011) were purchased from MedChem Express. SR8278 was synthesized and purified to over 98% purity by WuXiAppTec or Tocris.

### siRNA transfection.

siRNAs for gene knockdown were purchased from Dharmacon. The siRNA sequences against β-catenin/*CTNNB1* were AGCUGAUAUUGAUGGACAG for si*CTNNB1*-1 and GAUGGACUUUCCAGUCGUA for si*CTNNB1*-2. siRNA sequences against REV-ERBα/*NR1D1* were GCGCUUUGCUUCGUUGUUCAACUU for si*NR1D1*-1 and GCUGGCAUGUCCUAUGAACAUUU for si*NR1D1*-2. siRNA sequences against *EZH2* were GCUGAAGCCUCAAUGUUUA for si*EZH2*-1 and UAACGGUGAUCACAGGAUA for si*EZH2*-2; siRNA sequences against *EED* were CAACAGAGUUACCUUGUAU for si*EED*-1 and GGAUCUAGAGGCAUAAUUA for si*EED*-2. The siRNA control sequence CAGUCGCGUUUGCGACUGG was used as a nontargeting control. Transfections were performed with Opti-MEM (Invitrogen, Thermo Fisher Scientific) and Dharmafectin#1 (Dharmacon) following the manufacturer’s instructions.

### Overexpressed lentivirus production and infection.

For β-catenin overexpression (OE), lentiviral particles were produced in 293T cells after cotransfection with pLV–β-catenin deltaN90 lentivirus vector (Addgene, catalog 36985), psPAX2, and pMD2.G in 10 cm dishes. Cells were plated at 2 × 10^5^ cells per well in 6-well plates. Sixteen hours later, 1 mL virus-containing supernatant with 10 ng polybrene was added to the cells. After 4–6 hours, the medium was changed to regular medium and cultured with 20 μg/mL neomycin antibiotics–based selection for 7 days to generate cells stably expressing β-catenin deltaN90. REV-ERBα OE lentivirus and cells were prepared as described previously (42).

### qRT-PCR and Western blot analysis.

Total RNA was isolated from cells or xenograft tumors using TRIzol reagent (Invitrogen, Thermo Fisher Scientific, catalog 15596026). Total RNA (1 μg) was reverse-transcribed to cDNA using qScript cDNA SuperMix (Quantabio, catalog 95047-025). Next, qRT-PCR was performed using SYBR Green Master Mix (Applied Biosystems, catalog A25742) and gene specific primers. The PCR was run on a CFX96 connect Real-Time PCR system (Bio-Rad). The *GAPDH* gene transcript was used for normalization. The 2-ΔΔCt method was used to obtain relative quantifications. The experiments were performed at least 3 times, with data presented as mean values ± SD. The primers are listed in Supplemental Table 1. Cell lysates were analyzed by immunoblotting with antibodies specifically recognizing the proteins listed in Supplemental Table 4.

### Tumor sphere formation.

Tumor sphere formation was performed with C4-2B cells treated with Taz and EED226 and combined with Enz. A total of 500 cells were seeded into 6-well plates of ultra-low attachment and cultured for 10 days in serum-free Advanced DMEM/F-12 medium (Gibco, Thermo Fisher Scientific, catalog 12634010) supplemented with 2% B27 (Thermo Fisher Scientific, catalog A3582801), 20 ng/mL human epidermal growth factor (hEGF) (MilliporeSigma, catalog E5036), 10 ng/mL human insulin (Invitrogen, Thermo Fisher Scientific, catalog RP-10908), and 10 ng/mL basic fibroblast growth factor (PeproTech, catalog 100-18B). Tumor spheres were monitored and counted under a phase-contract microscope using a ×10 lens.

### Flow cytometry.

Cells were incubated with dissociation buffer (3 mM EDTA in 1× PBS) at 37°C for 10 minutes before being harvested using cell scrapers and filtered through 40 μm nylon cell strainers. Single-cell suspensions were subject to centrifugation at 500*g*. Cell pellets were resuspended in flow cytometry buffer (3 mM EDTA, 0.5% BSA in 1× PBS) containing anti-CD44, APC-conjugated (BioLegend, catalog 397506, dilution 1:20), and anti-CD133, PE-conjugated (BD, catalog 566593, dilution 1:20) antibodies and incubated for 1 hour at 4°C. Isotype antibodies were used as a negative control. Cells were washed twice with flow cytometry buffer and analyzed on a FACS system (BD Biosciences, BD Accuri C6 plus). Data were analyzed using FlowJo software (version 10.4.2).

### Statistics.

Statistical analyses were performed using GraphPad Prism 8.0 (GraphPad Software). All statistical details of experiments are included in the figure legends or in Methods section. All quantitative data are presented as the mean ± SEM unless otherwise indicated. Statistical tests were performed by a 2-tailed Student’s test, and a *P* value of less than 0.05 was considered significant. Details for the statistical test performed for individual figures are provided in the respective figure legends.

### Study approval.

All animal procedures were carried out in accordance with NIH guidelines and approved by the IACUC of UCD. Prostate cancer tumor specimens were collected by the Biorepository Shared Resource at the Medical Center of UCD after informed consent was obtained and delivered to this study without patient identification information.

### Data availability.

All raw data and processed information for the ChIP-seq, RNA-seq, and scRNA-seq experiments in this study have been deposited in the Gene Expression Omnibus (GEO) database (GSE294887, GSE325232, and GSE294966). Raw data used to prepare graphs in the figures are provided in the Supporting Data Values file.

## Author contributions

HWC and YY conceived the project. HWC, YY, X Zhang, VBV, HZ, X Zheng, SG, CZC, and ADB designed the research, performed the experiments, analyzed the data, and/or interpreted the results. XC, EC, ACG, MADE, AZ, PNL, and HJK provided resources. HWC, YY, HB, VBV, RME, HJK, and AZ wrote and/or edited the manuscript. HWC supervised the project. The order of authors for co-first authorship was determined based primarily on the relative contributions of data presentation and manuscript writing.

## Conflict of interest

The authors have declared that no conflict of interest exists.

## Funding support

This work was supported, in whole or in part, by grants from the NIH and other funding agencies. In accordance with the NIH Public Access Policy, the final peer-reviewed manuscript will be submitted to PubMed Central upon acceptance.

- NIH R01 CA259081 and NIH R01 CA224900, to HWC.
- The US Department of Defense (DoD) PCRP PC200522, to HWC.
- The US Department of Veterans Affairs, Office of Research and Development BL&D 2I01 BX004271, to HWC.
- NIH R01 CA292660, to XC.
- DoD PCRP Early Career Investigator Award W81XWH2210197, National Cancer Center Postdoctoral Fellowship Award, and Prostate Cancer Foundation Young Investigator Award 23YOUN15, to VBV.
- NIH/NCI DF/HCC SPORE P50 CA272390-01, to HB.
- NIH/NCI Cancer Center Support Grant NCI P30CA093373 to UCD Comprehensive Cancer Center (UCDCCC) Genomics Shared Resources (GSR).

## Supplementary Material

Supplemental data

Unedited blot and gel images

Supplemental table 1

Supplemental table 2

Supplemental table 3

Supplemental table 4

Supporting data values

## Version 1 03/17/2026

In-Press Preview

## Version 2 05/01/2026

Electronic publication
