# Supplementary material for: Targeting Wnt/β-catenin and circadian regulator restores PRC2/EZH2-controlled chromatin bivalency and suppresses cell state diversity
Source: J Clin Invest. 2026 Mar 17;136(9):e200260. doi: 10.1172/JCI200260 (PMC13132380; doi:10.1172/JCI200260)
Supplement: Supplemental data [file jci-136-200260-s036.pdf]

Supplemental information

Targeting Wnt/β-Catenin and circadian regulator restores PRC2/EZH2 controlled chromatin bivalency and suppresses cell state diversity

Yatian Yang[1,15], Xiong Zhang[1,15], Varadha Balaji Venkadakrishnan[2,3], Hongye Zou[1], Xingling Zheng[1], Shiyao Guo[1], Christopher Z. Chen[4], Alexander D. Borowsky[5], Eva Corey[6], Ronald M. Evans[7], Allen C. Gao[8], Marc A. Dall'Era[8], Amina Zoubeidi[9,10], Primo N. Lara[11,12], Hsing-Jien Kung[1], Xinbin Chen[13], Himisha Beltran[2,3], Hong-Wu Chen[1,12,14*]

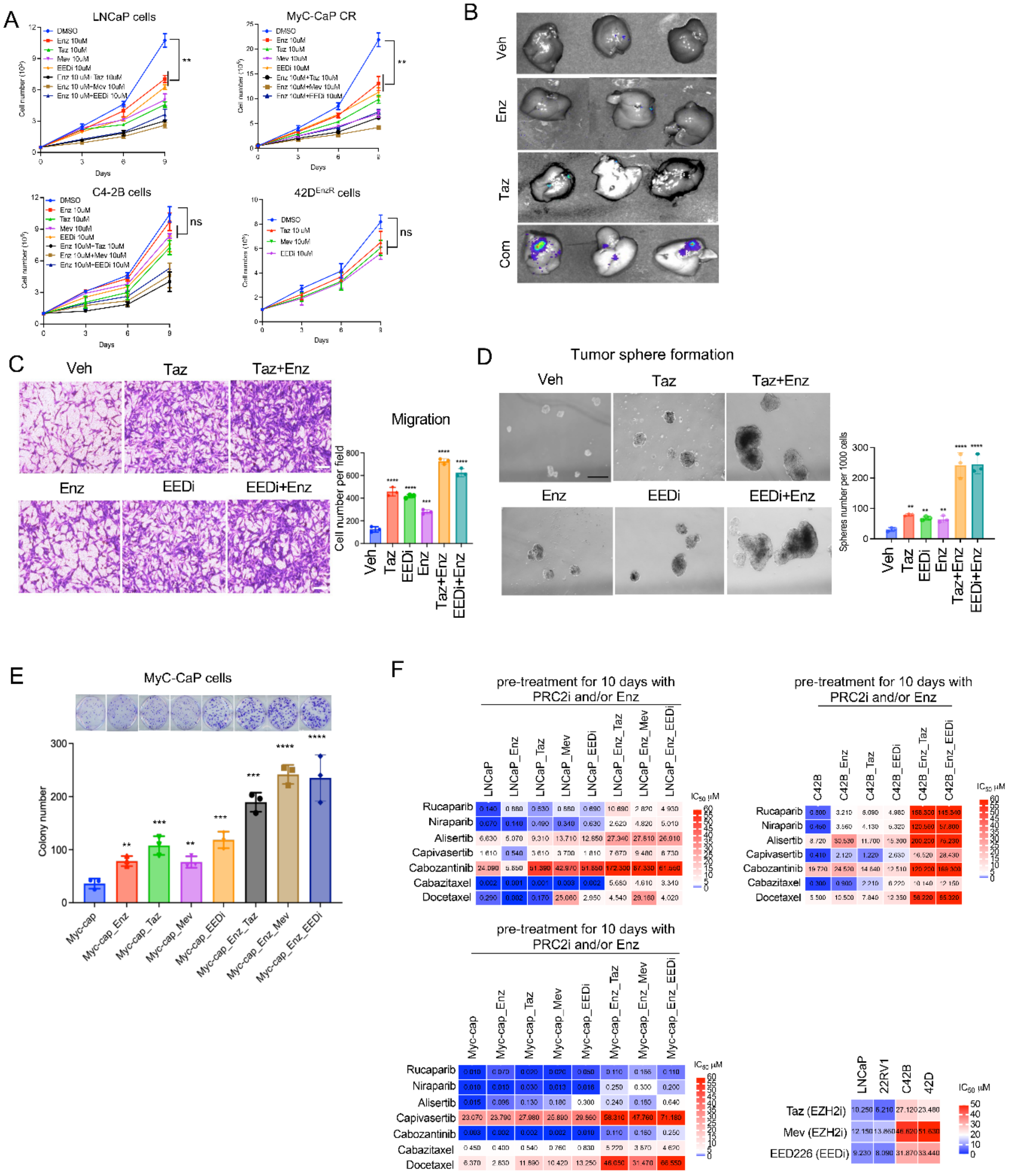

Supplemental Figure 1 Therapeutic targeting of PRC2/EZH2 and AR increases drug resistance and promotes tumor metastasis.

A. Cell growth curve of LNCaP, MyC-CaP, C4-2B and $42D^{EnzR}$ cells treated with indicated compounds. ** $p < 0.01$.

B. Representative images of livers with metastases detected by bioluminescence live imaging. The livers were dissected from mice carrying orthotopic MyC-CaP tumors and receiving indicated treatments for 14 days.

C. Representative images of migrated cells stained with crystal violet in C4-2B cells treated with indicated treatments for 48 h. ** $p < 0.01$; *** $p < 0.001$; **** $p < 0.0001$.

D. Tumor sphere formation of C4-2B cells treated with indicated treatments for 48 h. (Scale bar, 50 μm). ** $p < 0.01$; *** $p < 0.001$; **** $p < 0.0001$.

E. Cell colony formation of MyC-CaP cells pre-treated with indicated compounds for 10 days. ** $p < 0.01$; *** $p < 0.001$; **** $p < 0.0001$.

F. Heatmap of IC_{50} values of indicated compounds in indicated cells that were pretreated for 10 days in 10-cm plates treated by10 μM of Enz, EZH2 inhibitors, EED inhibitor, or their combination before they were re-plated in 96-well plates for treatments with the indicated drugs for 96 hrs. The IC_{50} values of PRC2 inhibitors in the different cells were measured with the cells treated for 96 hrs in 96-well plates (bottom right).

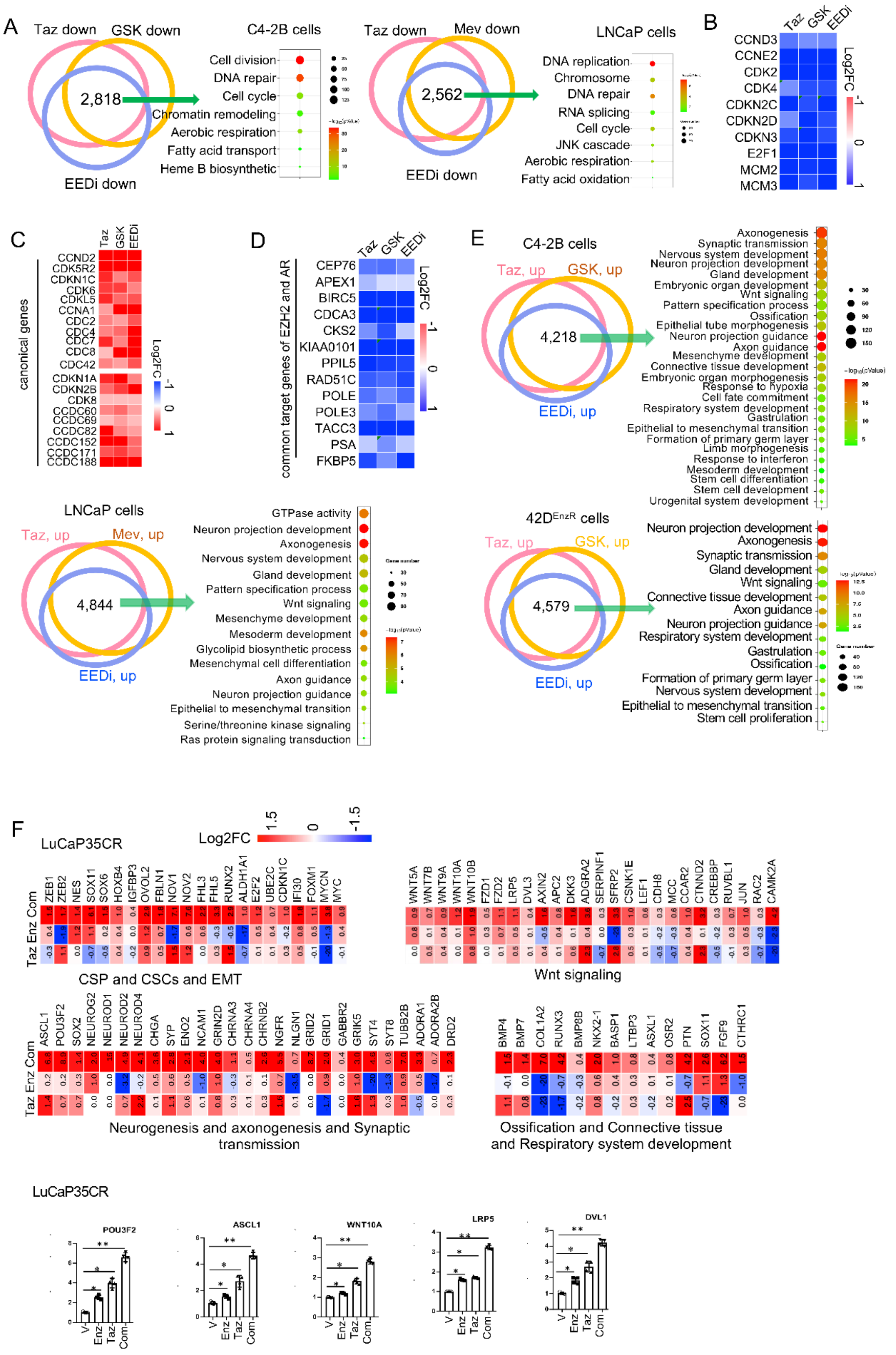

A
Taz down
GSK down
EEDi down
2,818
C4-2B cells
Cell division
DNA repair
Cell cycle
Chromatin remodeling
Aerobic respiration
Fatty acid transport
Heme B biosynthetic
Taz down
Mev down
EEDi down
2,562
LNCaP cells
DNA replication
Chromosome
DNA repair
RNA splicing
Cell cycle
JNK cascade
Aerobic respiration
Fatty acid oxidation
B
Taz
GSK
EEDi
CCND3
CCNE2
CDK2
CDK4
CDKN2C
CDKN2D
CDKN3
E2F1
MCM2
MCM3
Log2FC
C
canonical genes
CCND2
CDK5R2
CDKN1C
CDK6
CDKL5
CCNA1
CDC2
CDC4
CDC7
CDC8
CDC42
CDKN1A
CDKN2B
CDK8
CCDC60
CCDC69
CCDC82
CCDC152
CCDC171
CCDC188
D
common target genes of EZH2 and AR
CEP76
APEX1
BIRC5
CDCA3
CKS2
KIAA0101
PPIL5
RAD51C
POLE
POLE3
TACC3
PSA
FKBP5
E
C4-2B cells
Taz, up
GSK, up
EEDi, up
4,218
Axonogenesis
Synaptic transmission
Nervous system development
Neuron projection development
Gland development
Embryonic organ development
Wnt signaling
Pattern specification process
Ossification
Epithelial tube morphogenesis
Neuron projection guidance
Axon guidance
Mesenchyme development
Connective tissue development
Embryonic organ morphogenesis
Response to hypoxia
Cell fate commitment
Respiratory system development
Gastrulation
Epithelial to mesenchymal transition
Formation of primary germ layer
Limb morphogenesis
Response to interferon
Mesoderm development
Stem cell differentiation
Stem cell development
Urogenital system development
LNCaP cells
Taz, up
Mev, up
EEDi, up
4,844
GTPase activity
Neuron projection development
Axonogenesis
Nervous system development
Gland development
Pattern specification process
Wnt signaling
Mesenchyme development
Mesoderm development
Glycolipid biosynthetic process
Mesenchymal cell differentiation
Axon guidance
Neuron projection guidance
Epithelial to mesenchymal transition
Serine/threonine kinase signaling
Ras protein signaling transduction
42D^EnzR cells
Taz, up
GSK, up
EEDi, up
4,579
Neuron projection development
Axonogenesis
Synaptic transmission
Gland development
Wnt signaling
Connective tissue development
Axon guidance
Neuron projection guidance
Respiratory system development
Gastrulation
Ossification
Formation of primary germ layer
Nervous system development
Epithelial to mesenchymal transition
Stem cell proliferation
F
LuCaP35CR
Log2FC
1.5
0
-1.5
Taz Enz Com
CSP and CSCs and EMT
Wnt signaling
Neurogenesis and axonogenesis and Synaptic transmission
Ossification and Connective tissue and Respiratory system development
LuCaP35CR
POU3F2
ASCL1
WNT10A
LRP5
DVL1
V
Enz
Taz
Com

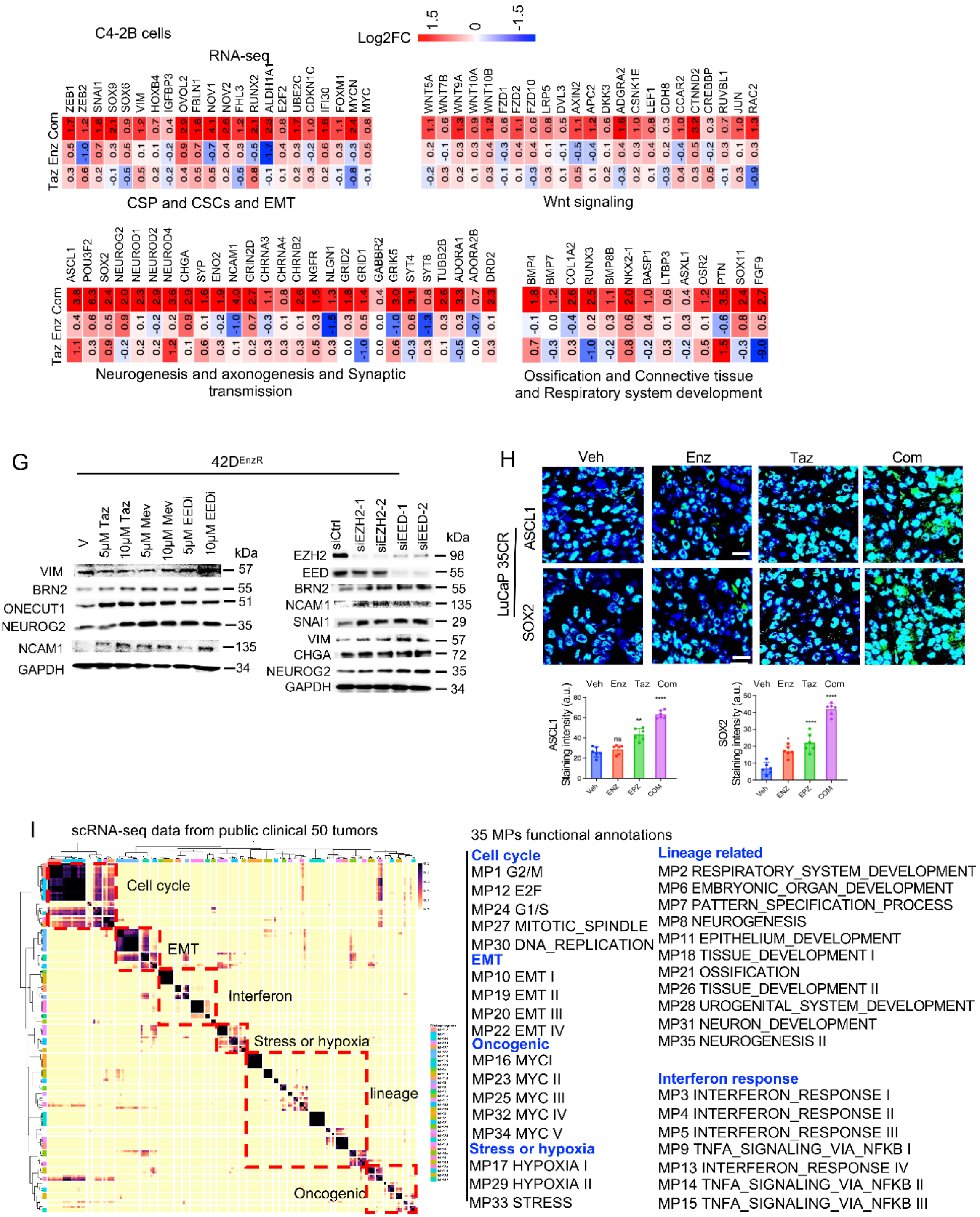
C4-2B cells
RNA-seq
Log2FC
1.5
0
-1.5
Taz Enz Com
CSP and CSCs and EMT
Wnt signaling
Neurogenesis and axonogenesis and Synaptic transmission
Ossification and Connective tissue and Respiratory system development
G
42D EnzR
V
5μM Taz
10μM Taz
5μM Mev
10μM Mev
5μM EEDi
10μM EEDi
kDa
VIM
BRN2
ONECUT1
NEUROG2
NCAM1
GAPDH
siCtrl
siEZH2-1
siEZH2-2
siEED-1
siEED-2
EZH2
EED
SNAI1
CHGA
H
Veh
Enz
Taz
Com
LuCaP 35CR
ASCL1
SOX2
Staining intensity (a.u.)
I
scRNA-seq data from public clinical 50 tumors
Cell cycle
EMT
Interferon
Stress or hypoxia
lineage
Oncogenic
35 MPs functional annotations
Cell cycle
MP1 G2/M
MP12 E2F
MP24 G1/S
MP27 MITOTIC_SPINDLE
MP30 DNA_REPLICATION
EMT
MP10 EMT I
MP19 EMT II
MP20 EMT III
MP22 EMT IV
Oncogenic
MP16 MYCI
MP23 MYC II
MP25 MYC III
MP32 MYC IV
MP34 MYC V
Stress or hypoxia
MP17 HYPOXIA I
MP29 HYPOXIA II
MP33 STRESS
Lineage related
MP2 RESPIRATORY_SYSTEM_DEVELOPMENT
MP6 EMBRYONIC_ORGAN_DEVELOPMENT
MP7 PATTERN_SPECIFICATION_PROCESS
MP8 NEUROGENESIS
MP11 EPITHELIUM_DEVELOPMENT
MP18 TISSUE_DEVELOPMENT I
MP21 OSSIFICATION
MP26 TISSUE_DEVELOPMENT II
MP28 UROGENITAL_SYSTEM_DEVELOPMENT
MP31 NEURON_DEVELOPMENT
MP35 NEUROGENESIS II
Interferon response
MP3 INTERFERON_RESPONSE I
MP4 INTERFERON_RESPONSE II
MP5 INTERFERON_RESPONSE III
MP9 TNFA_SIGNALING_VIA_NFKB I
MP13 INTERFERON_RESPONSE IV
MP14 TNFA_SIGNALING_VIA_NFKB II
MP15 TNFA_SIGNALING_VIA_NFKB III

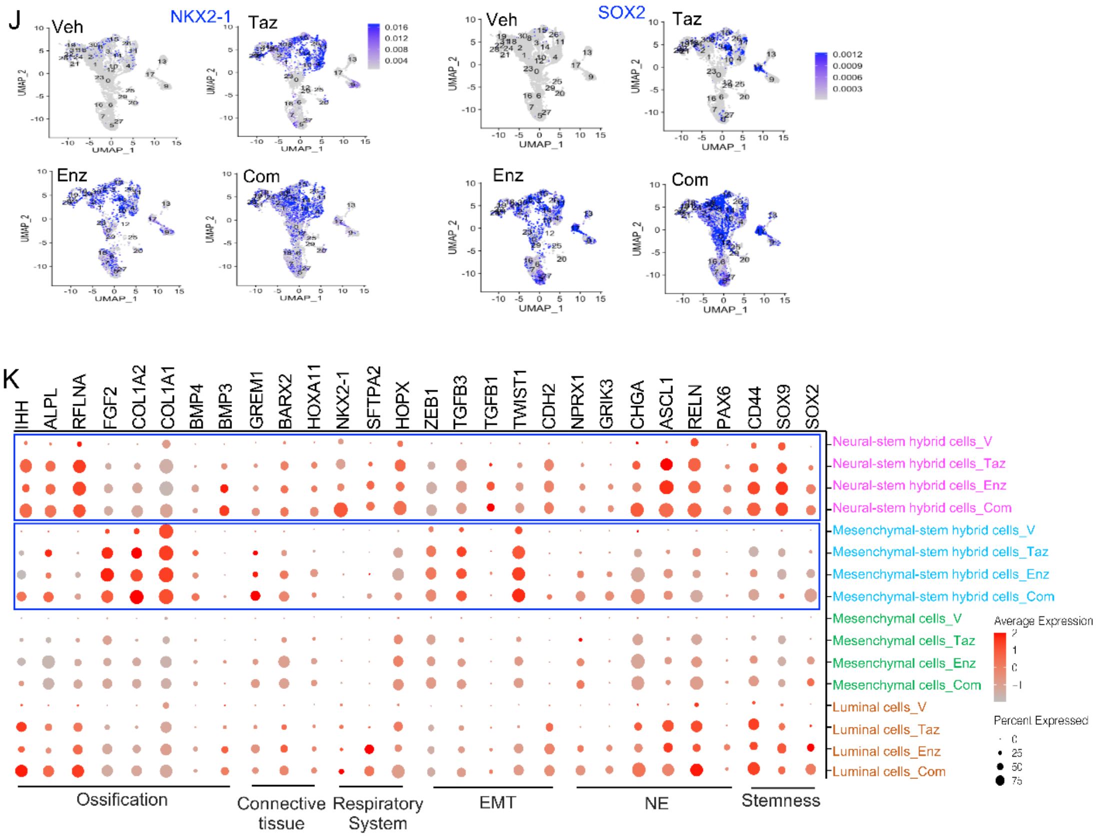

Supplemental Figure 2 Therapeutic targeting of PRC2/EZH2 and AR promotes cell state diversity.

A. Venn diagram of genes downregulated by Taz, GSK126 and Mev for 48 h (FC>=1.5). Bubble plot of top GO programs of 2,818 genes in C4-2B cells and 2,562 genes in LNCaP cells.

B. Heatmap of indicated gene expression in C4-2B cells treated as indicated for 48 h.

C. Heatmap of indicated gene expression in C4-2B cells treated as indicated for 48 h.

D. Heatmap of indicated gene expression changes in C4-2B cells treated as indicated for 48 h.

E. Venn diagram of genes upregulated by Taz (EPZ6438), GSK126, Mev (PF06821497) and EEDi (EED226) in C4-2B, LNCaP and 42D^{EnzR} cells for 48 h (FC>=1.5). Bubble plot of top GO programs of 4,218, 4,884, 4,579 genes and Z-score of each program.

F. Heatmaps of indicated gene expression changes when compared to vehicle in LuCaP 35CR tumors in mice treated as indicated for 10 days and C4-2B cell treated as indicated for 48h, and bar graphs of RT-qPCR analysis of indicated genes in LuCaP 35CR tumors (middle panels). ** $p < 0.01$; *** $p < 0.001$; **** $p < 0.0001$.

G. Western blotting of indicated proteins in 42D^{EnzR} cells treated with indicated compounds or siRNAs for 72 h.

H. Representative images of confocal microscopy analysis of indicated LP protein expression in LuCaP 35CR tumors in mice treated as indicated. Scale bars, 50 μm. Bar graphs display ImageJ-analyzed immunofluorescence intensity from 7 randomly selected fields per tumor. a.u., arbitrary unit. ** $p < 0.01$; *** $p < 0.001$; **** $p < 0.0001$.

I. Left. Heatmap showing similarity indices for comparison of 35 robust MPs identified based on their top 90 genes. Programs are ordered by clustering and grouped into MPs and families of MPs with related functions (marked by red dashed lines); MP families are numbered and labelled. Right. List of all MPs and their separation into 6 MP families.

J. UMAP visualization of the different subtypes of epithelial cells colored by a gradient of indicated gene expression. The minimum score is indicated by light grey and the maximum score is indicated by blue.

K. Bubble plot visualization of the different subtypes of epithelial cells colored by a gradient of indicated gene expression. The minimum score is indicated by light grey and the maximum score is indicated by red.

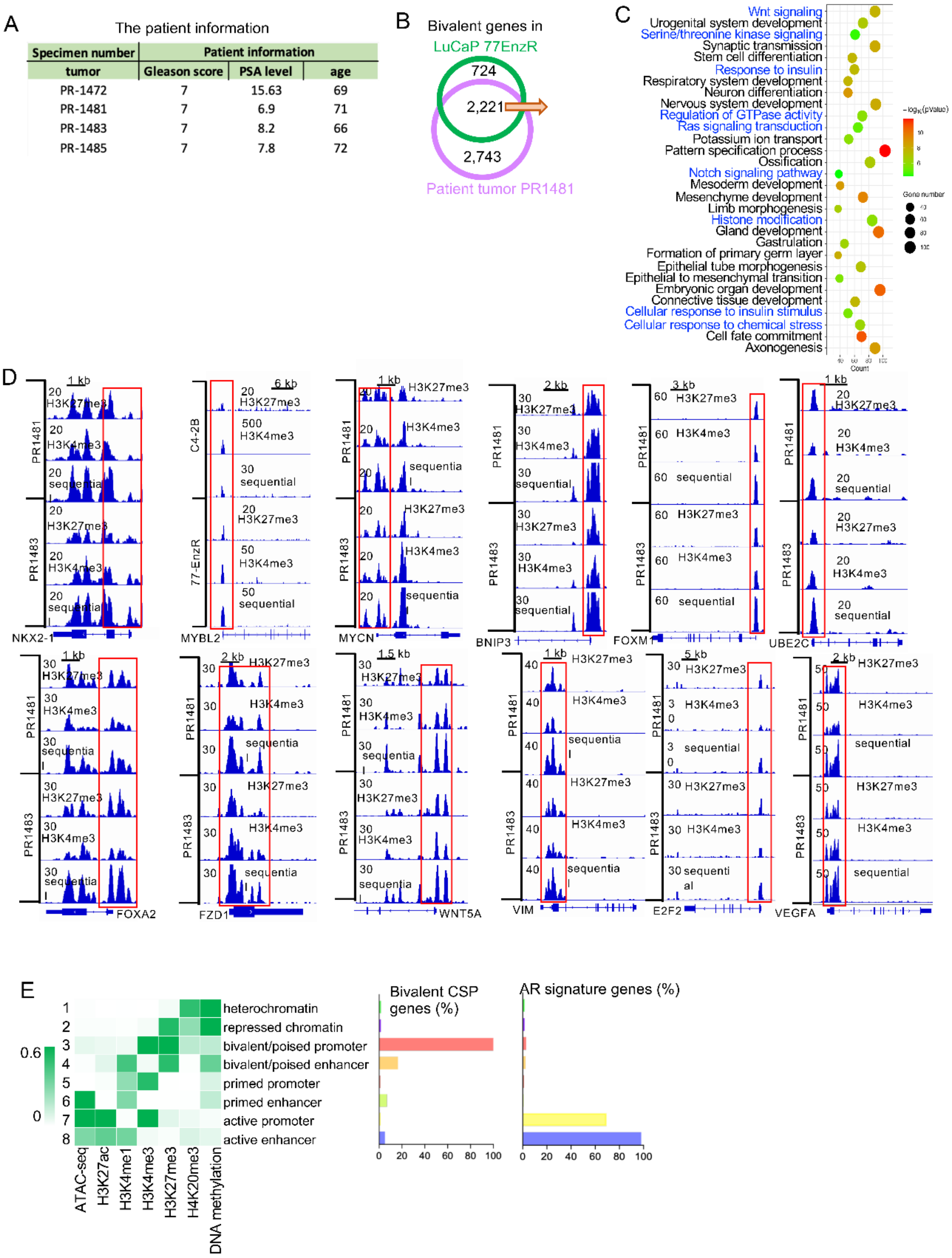

Specimen number	Patient information		
tumor	Gleason score	PSA level	age
PR-1472	7	15.63	69
PR-1481	7	6.9	71
PR-1483	7	8.2	66
PR-1485	7	7.8	72

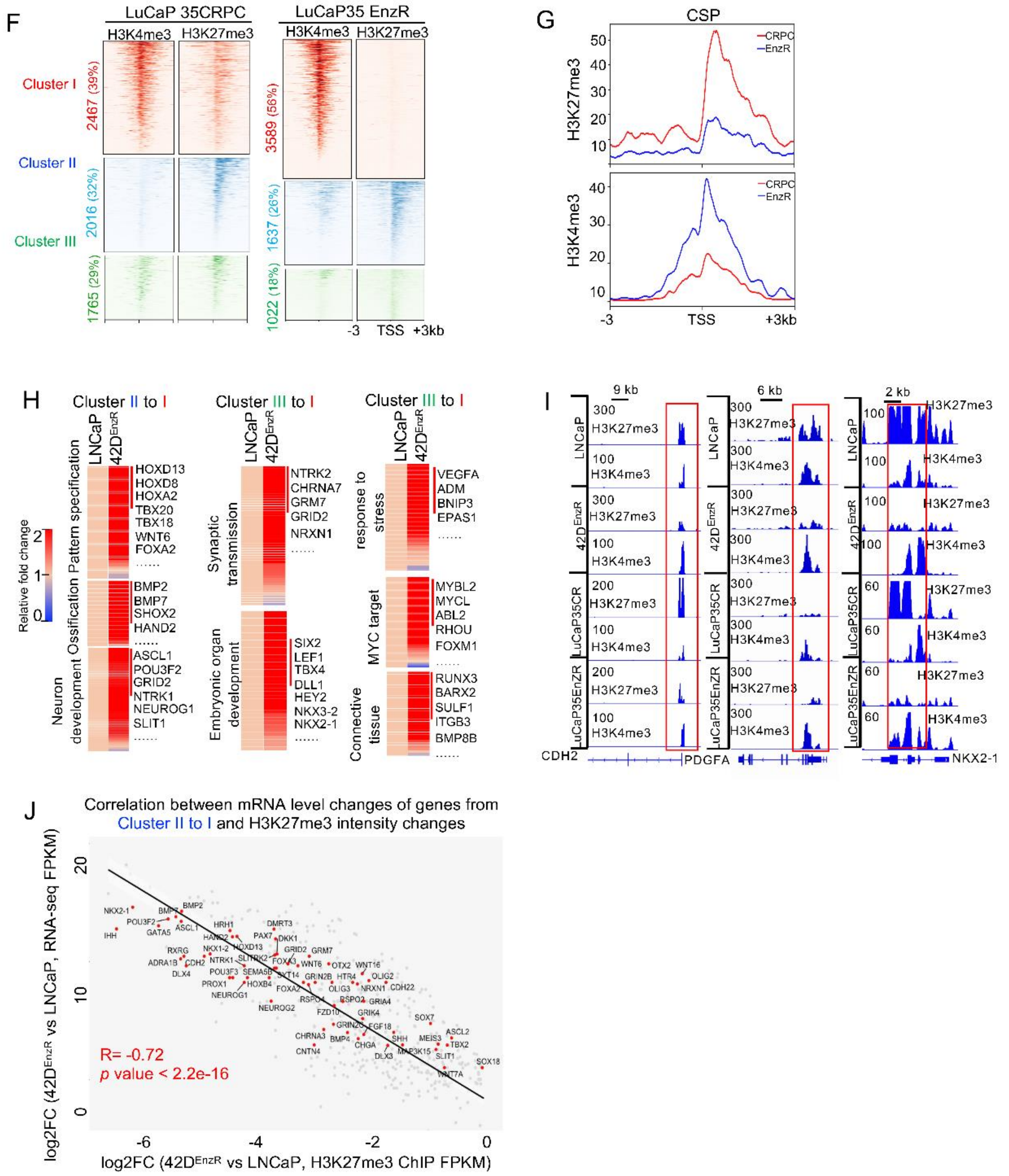

Supplemental Figure 3 Induction of cell state diversity is associated with alteration of chromatin bivalency.

A. Patients information of fresh tumors with bivalent promoters identified.

B. Venn diagram of overlapped bivalent genes (2,221) in indicated tumors.

C. Bubble plot of GO programs enriched by overlapped bivalent genes (2,221) in B.

D. IGV snapshots of sequential or separate ChIP-seq of H3K27me3 and H3K4me3 at representative CSP genes in indicated patient tumors.

E. Left, chromatin state analysis by ChromHMM of tumor cells of prostate cancer. Green shadings depict the average intensity of a particular mark across each chromatin state. Right, the percentage (%) of indicated gene sets distributed in the different chromatin states.

F. K-means analysis of bivalent promoters and heatmaps of H3K4me3 and H3K27me3 ChIP-seq signal intensity within +/-3 kb windows around TSS at cluster I (H3K4me3-high, with intensity of H3K4me3 higher than H3K27me3 fold change (FC) $\geq$1.5), cluster II (H3K27me3-high, with intensity of H3K27me3 higher than H3K4me3 FC $\geq$1.5), and cluster III (H3K4me3/K27me3-equal, with intensity of H3K27me3 equal to that of H3K4me3 $|FC|<1.5$ in indicated models.

G. Signal profiles of H3K4me3 and H3K27me3 ChIP-seq signal intensity within +/-3 kb windows around TSS at bivalent genes of indicated programs.

H. Heatmaps of relative fold changes in gene expression detected by RNA-seq in indicated cells, based on their chromatin bivalency changes from cluster II or III to I.

I. IGV snapshots of H3K27me3 and H3K4me3 ChIP-seq at representative genes in indicated cells and PDX models.

J. Scatter plot of $\log_2$FC in H3K27me3 mark intensity between $42D^{EnzR}$ and LNCaP cells at all bivalent promoters and $\log_2$FC in bivalent gene transcript levels between $42D^{EnzR}$ and LNCaP cells. Pearson's correlation scores and associated *p* value are indicated.

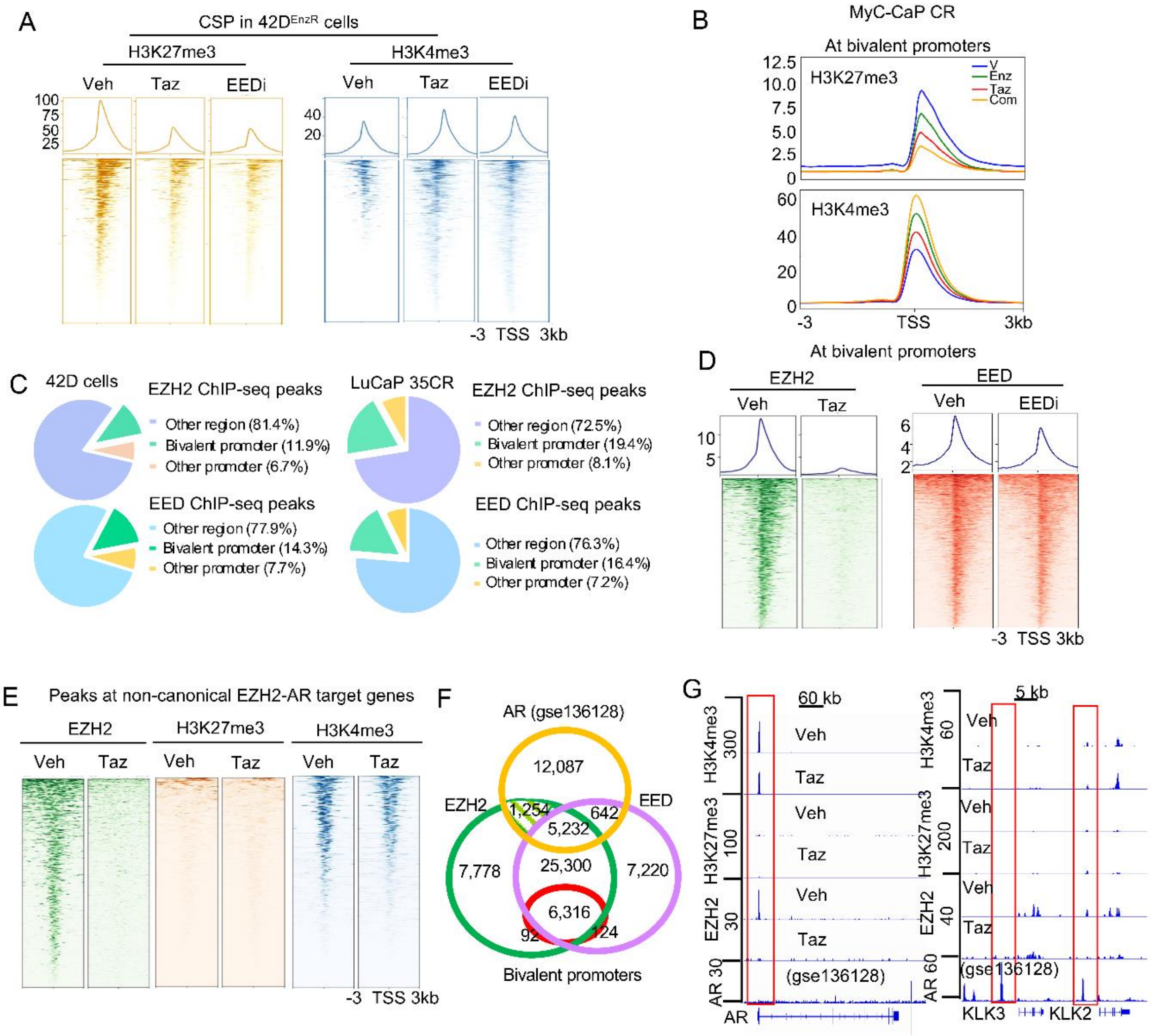
A
CSP in 42D^EnzR cells
H3K27me3
H3K4me3
Veh
Taz
EEDi
-3 TSS 3kb
B
MyC-CaP CR
At bivalent promoters
H3K27me3
H3K4me3
V
Enz
Taz
Com
-3
TSS
3kb
C
42D cells
EZH2 ChIP-seq peaks
Other region (81.4%)
Bivalent promoter (11.9%)
Other promoter (6.7%)
EED ChIP-seq peaks
Other region (77.9%)
Bivalent promoter (14.3%)
Other promoter (7.7%)
LuCaP 35CR
EZH2 ChIP-seq peaks
Other region (72.5%)
Bivalent promoter (19.4%)
Other promoter (8.1%)
EED ChIP-seq peaks
Other region (76.3%)
Bivalent promoter (16.4%)
Other promoter (7.2%)
D
At bivalent promoters
EZH2
EED
Veh
Taz
EEDi
E
Peaks at non-canonical EZH2-AR target genes
EZH2
H3K27me3
H3K4me3
F
AR (gse136128)
12,087
EZH2
1,254
642
EED
5,232
25,300
7,778
7,220
6,316
92
124
Bivalent promoters
G
60 kb
5 kb
(gse136128)
AR
KLK3
KLK2

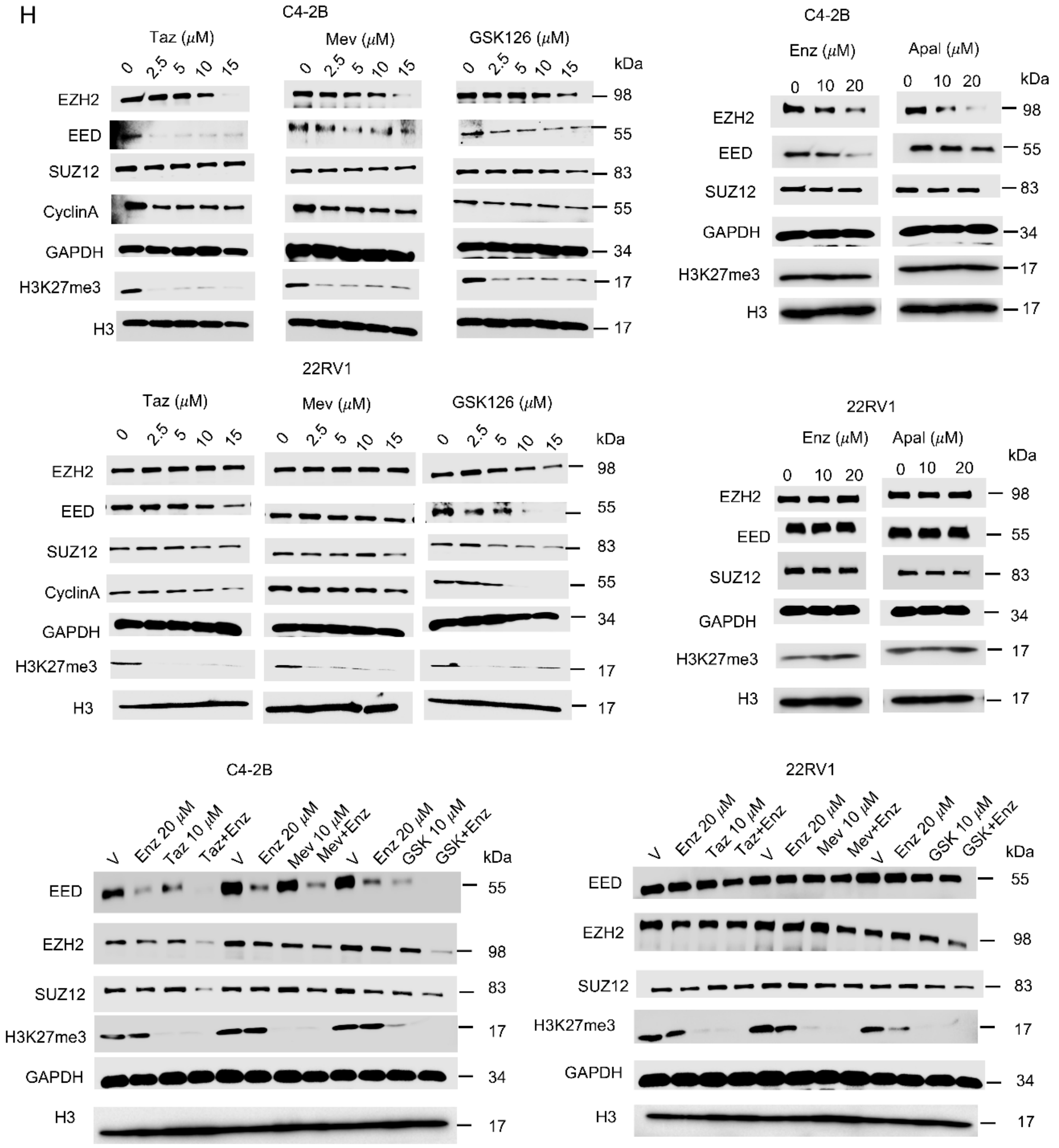

Supplemental Figure 4 Co-targeting of PRC2 and AR effectively disrupts the functional balance of PRC2 and KMT2B in bivalency maintenance.

A. Heatmaps and signal profiles of H3K27me3 and H3K4me3 ChIP-seq signal intensity within +/- 3 kb windows at TSS at LP programs in $42D^{EnzR}$ cells treated as indicated for 24 h.

B. Signal profile of H3K27me3 and H3K4me3 ChIP-seq signal intensity within +/- 3 kb windows at TSS at all bivalent gene promoters in MyC-CaP CR mouse tumors treated as indicated for 10 days.

C. Pie chart of genomic location distributions of EZH2 and EED ChIP-seq peaks in C4-2B cells and LuCaP 35CR tumors.

D. Heatmaps and signal profiles of EZH2 and EED ChIP-seq signal intensity within +/- 3 kb windows at TSS at all bivalent gene promoters in $42D^{EnzR}$ cells treated as indicated for 24 h.

E. Heatmaps of EZH2, H3K27me3 and H3K4me3 ChIP-seq signal intensity within +/- 3 kb windows at TSS at non-canonical EZH2 target gene loci (1,254 as shown in F) in $42D^{EnzR}$ cells treated by 10 μM Taz or vehicle for 24 h.

F. Venn diagram of ChIP-seq peak overlaps between AR (GSE136128) and EZH2 or EED. Peaks (1,254) overlapped between AR and EZH2 only are defined as non-canonical EZH2 targets.

G. IGV snapshots of AR, EZH2, EED, H3K27me3 and H3K4me3 ChIP-seq at representative non-canonical EZH2 target genes in C4-2B cells treated by 10 μM Taz, 10 μM EEDi (EED226) or vehicle for 24 h.

H. Western blotting of indicated proteins in indicated cells treated with indicated treatments for 72 h.

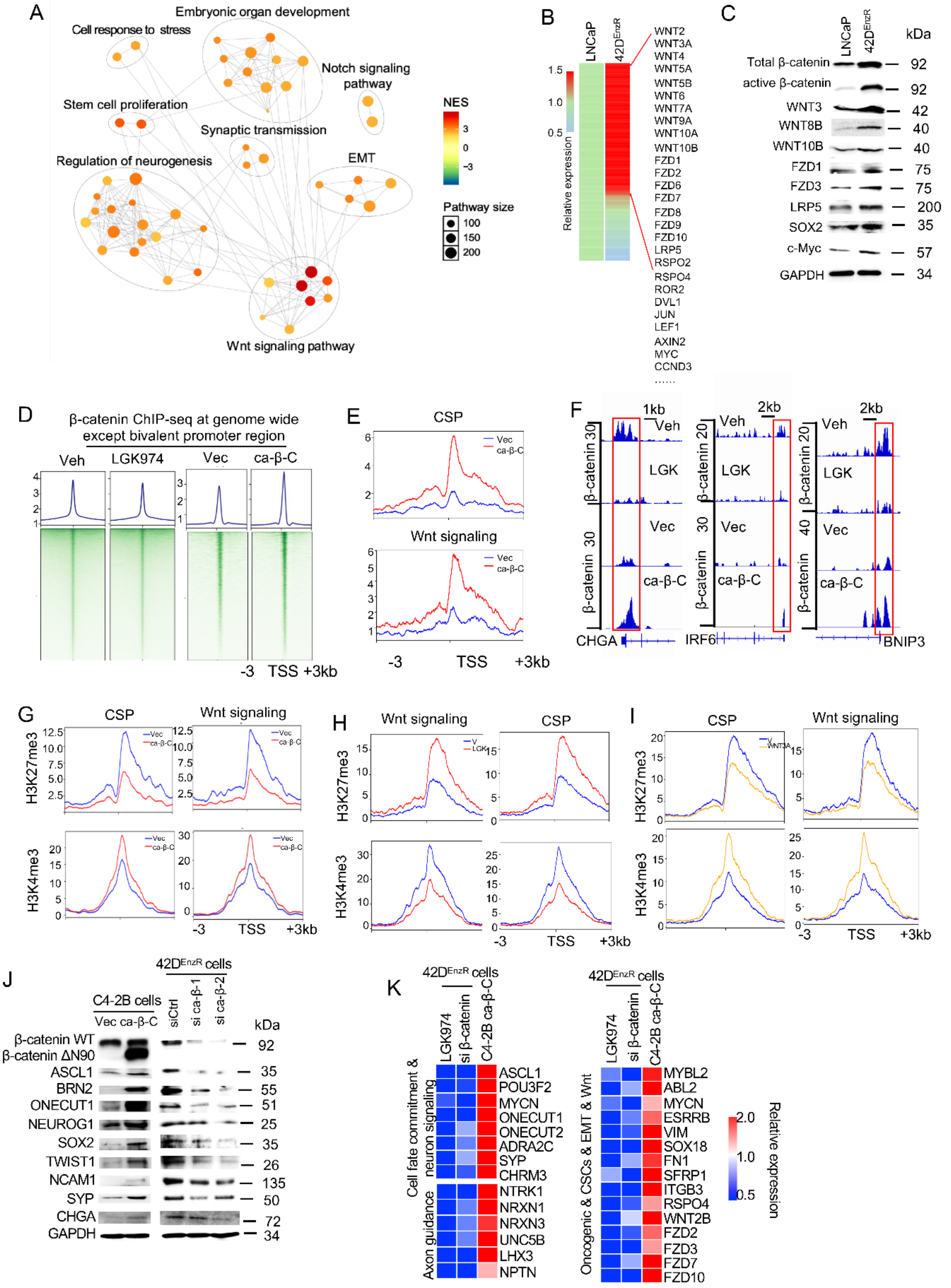
A
Embryonic organ development
Cell response to stress
Notch signaling pathway
Stem cell proliferation
Synaptic transmission
Regulation of neurogenesis
EMT
Wnt signaling pathway
NES
Pathway size
B
LNCaP
42D EnzR
Relative expression
WNT2
WNT3A
WNT4
WNT5A
WNT5B
WNT6
WNT7A
WNT9A
WNT10A
WNT10B
FZD1
FZD2
FZD6
FZD7
FZD8
FZD9
FZD10
LRP5
RSPO2
RSPO4
ROR2
DVL1
JUN
LEF1
AXIN2
MYC
CCND3
C
Total β-catenin
active β-catenin
WNT3
WNT8B
WNT10B
FZD1
FZD3
LRP5
SOX2
c-Myc
GAPDH
kDa
D
β-catenin ChIP-seq at genome wide except bivalent promoter region
Veh
LGK974
Vec
ca-β-C
-3 TSS +3kb
E
CSP
Wnt signaling
F
β-catenin
CHGA
IRF6
BNIP3
G
H3K27me3
H3K4me3
H
I
J
C4-2B cells
42D EnzR cells
β-catenin WT
β-catenin ΔN90
ASCL1
BRN2
ONECUT1
NEUROG1
SOX2
TWIST1
NCAM1
SYP
CHGA
GAPDH
K
Cell fate commitment & neuron signaling
Axon guidance
Oncogenic & CSCs & EMT & Wnt
Relative expression

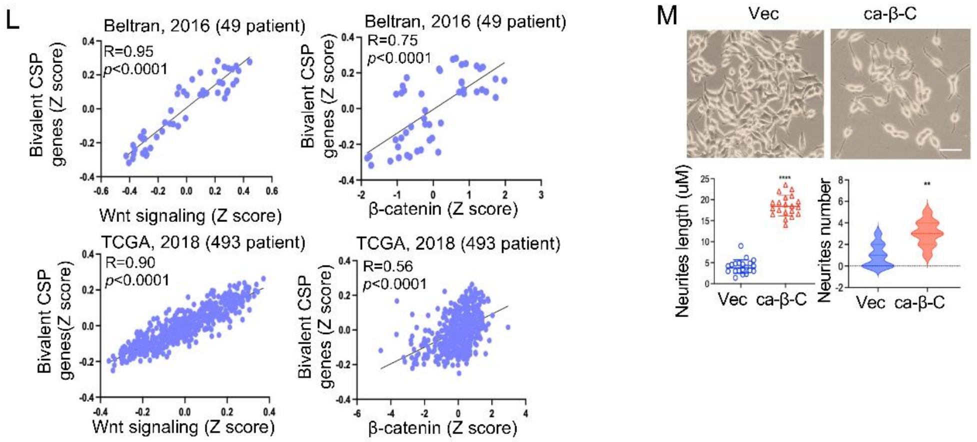

Supplemental Figure 5 Wnt/β-catenin signaling drives changes of chromatin bivalency.

A. Biological clustering of GO programs with genes associated with increased H3K4me3 and decreased H3K27me3 by Taz and Enz combination treatment of mice with LuCaP 35CR tumors for 10 days.

B. Heatmaps of $\log_2$ fold changes of Wnt signaling gene expression detected by RNA-seq in indicated cell lines.

C. Western blotting of indicated proteins in indicated cells.

D. Signal profiles and heatmaps of β-catenin ChIP-seq signal intensity within +/-3 kb windows around TSS at indicated regions in C4-2B cells with indicated treatments.

E. Signal profiles of β-catenin ChIP-seq peaks intensity within +/- 3 kb windows around the TSS of peaks at indicated programs in C4-2B cells with constitutive active β-catenin (ca-β-C)-expression.

F. IGV snapshots of indicated ChIP-seq at indicated bivalent genes.

G-I. Signal profiles of H3K4me3 and H3K27me3 ChIP-seq signal intensity within +/-3 kb windows around TSS at indicated programs in C4-2B cells treated with ca-β-C-expression (G), or with 10 µM LGK974 for 24 h (H), or 5 µM Wnt3a for 48 h (I).

J. Western blotting of indicated proteins in C4-2B cells with ca-β-C-expression and $42D^{EnzR}$ cells treated with indicated treatments for 72 h.

K. Heatmap of mRNA expression changes detected by RNA-seq in C4-2B cells with ca-β-C-expression and $42D^{EnzR}$ cells with indicated treatments for 48 h.

L. Expression correlations (Z score) between Wnt signaling or β-catenin and bivalent CSP genes in Beltran et al. (2016) and TCGA (2018) datasets and with each dot representing a patient tumor. Significance was evaluated by linear regression *t* test.

M. Number of cells with neurites-like protrusions and the protrusion length in C4-2B cell with or without ca-β-C-expression. Scale bar, 50 μm. ** $p < 0.01$; *** $p < 0.001$; **** $p < 0.0001$.

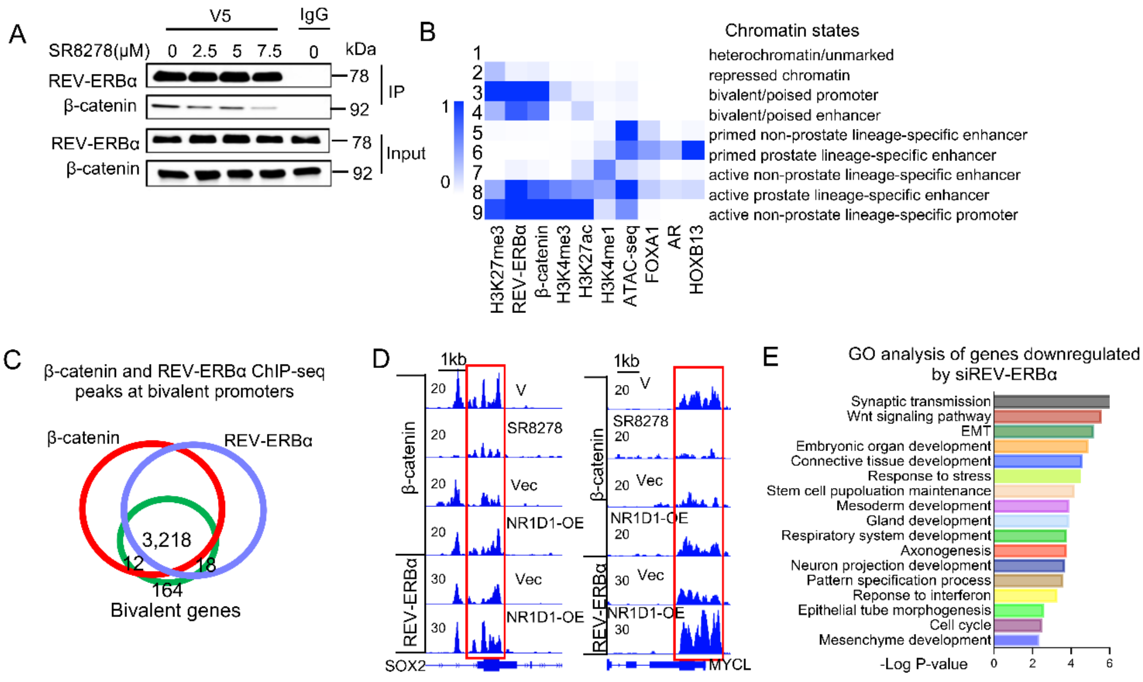

Supplemental Figure 6 REV-ERBα mediates Wnt/β-catenin function in driving changes of chromatin bivalency.

A. Co-immunoprecipitation analysis of REV-ERBα and β-catenin association in C4-2B cells overexpressing V5-REV-ERBα and treated with indicated concentrations of SR8278 for 24 h.

B. Chromatin state analysis by ChromHMM of C4-2B cells with nine epigenetic and TF ChIP-seq marks. Blue shadings depict the average intensity of a particular mark across each chromatin state.

C. Venn diagram of genes with REV-ERBα and β-catenin ChIP-seq peaks at bivalent promoters and bivalent genes.

D. IGV snapshots of indicated ChIP-seq at indicated bivalent genes.

E. GO analysis of downregulated genes by siREV-ERBα for 48 h in C4-2B cells with ca-β-C-expression.

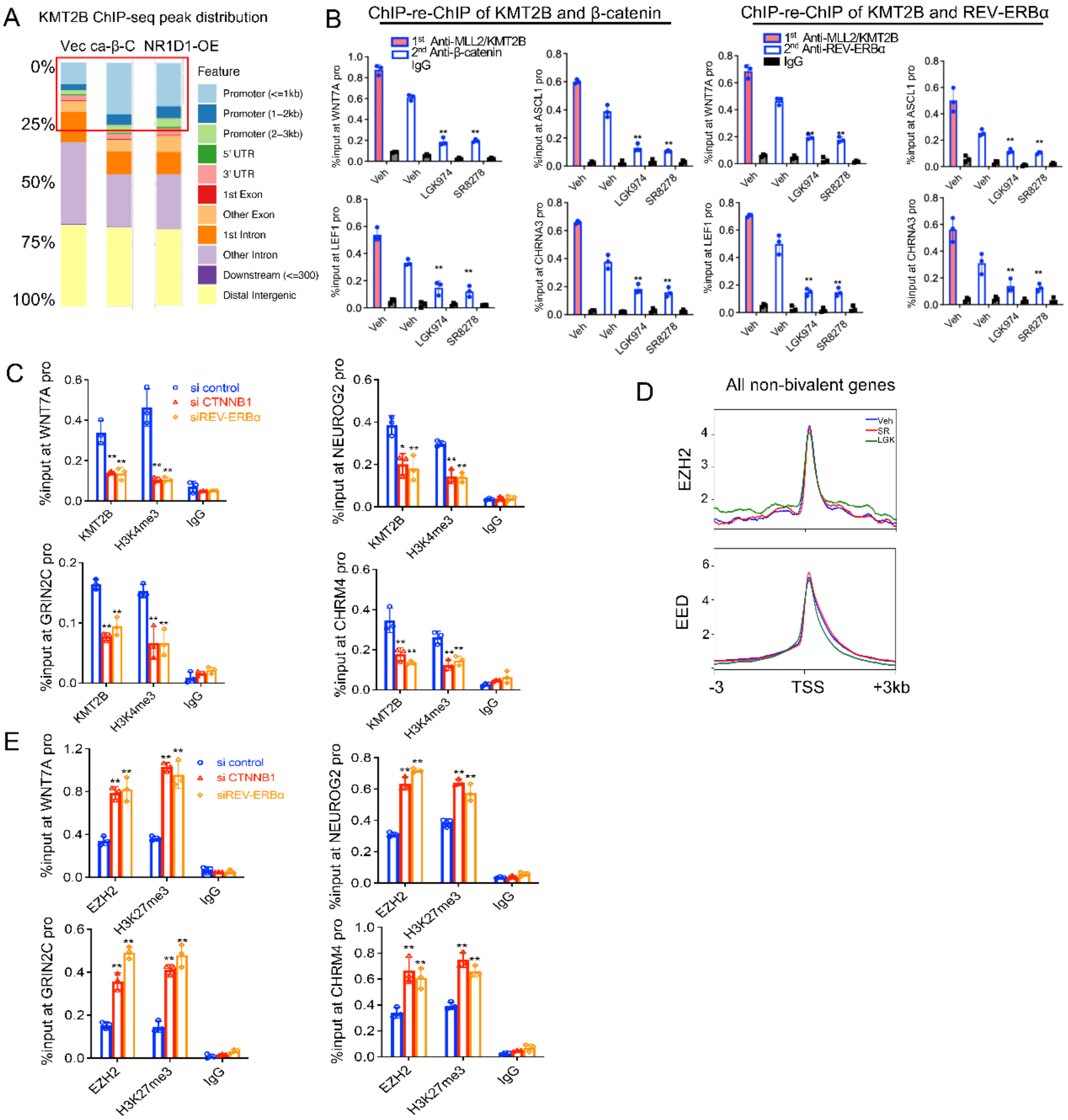

Supplemental Figure 7 Hyperactive Wnt/β-catenin and REV-ERBα promotes KMT2B function and antagonizes PRC2 in driving chromatin bivalency change.

A. KMT2B ChIP-seq peak genomic location distribution in C4-2B cells with ca-β-C-expression and REV-ERBα overexpression or vector control.

B. ChIP-re-ChIP qPCR analysis of KMT2B and β-catenin (left), and REV-ERBα (right) at promoters of indicated genes in $42D^{EnzR}$ cells treated by vehicle or 10 μM LGK974, 7.5 μM SR8278 for 24 h. (mean ± s. d., n = 3). ** $p < 0.01$.

C. ChIP-qPCR analysis of KMT2B and H3K4me3 at promoters of indicated genes in $42D^{EnzR}$ cells treated by si*CTNNB1* and siREV-ERBα for 24 h. (mean ± s. d., n = 3). ** $p < 0.01$.

D. Signal profiles of EZH2 and EED ChIP-seq within +/- 3 kb windows around TSS at non-bivalent gene promoters in C4-2B cells treated with 10 μM LGK974 and 7.5 μM SR8278 for 24 h.

E. ChIP-qPCR analysis of EZH2, H3K27me3 at indicated genes in $42D^{EnzR}$ cells treated by si*CTNNB1* and si*NR1D1*/REV-ERBα for 24 h. (mean ± s. d., n = 3). ** $p < 0.01$.

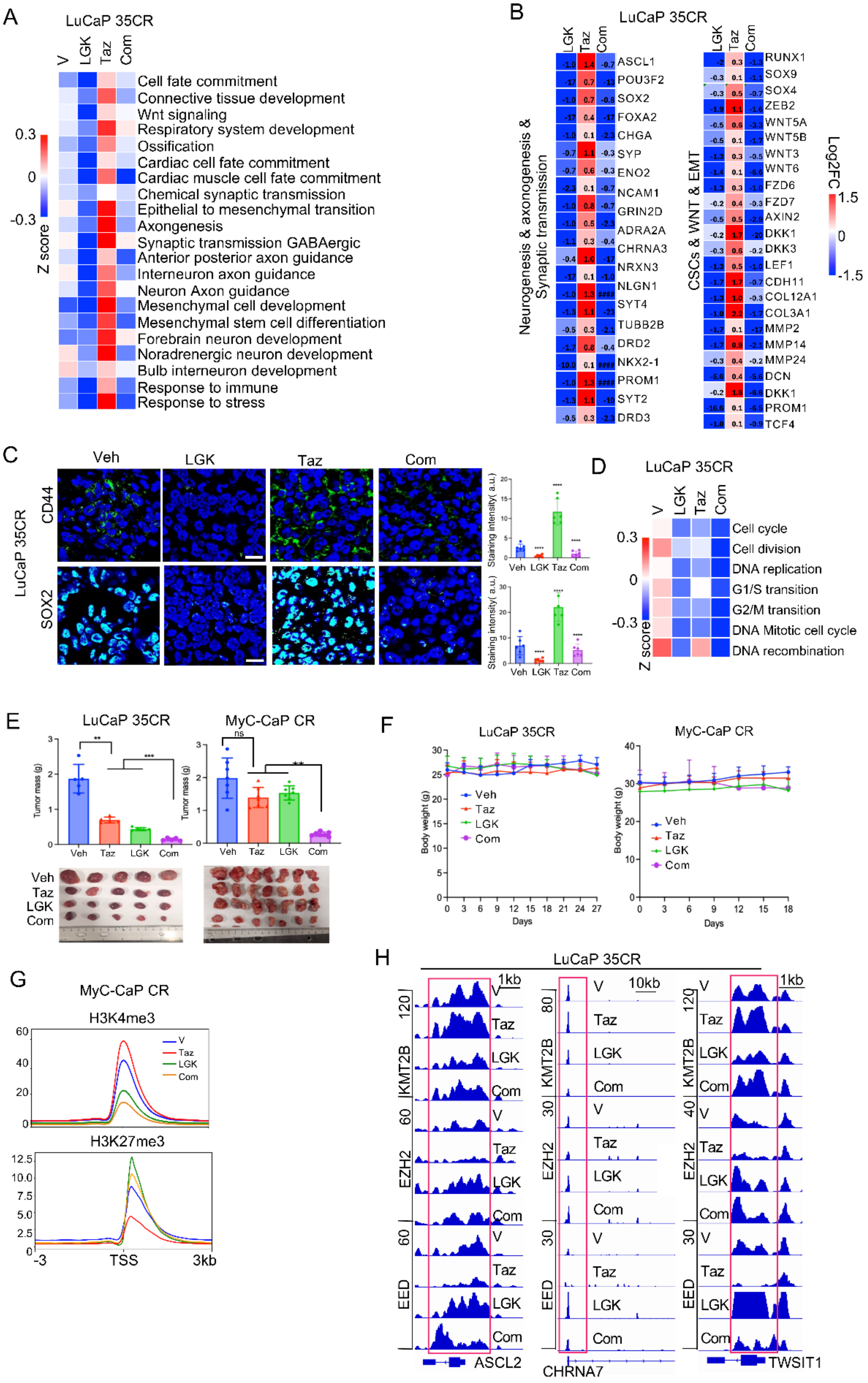

A
LuCaP 35CR
V
LGK
Taz
Com
0.3
0
-0.3
Z score
Cell fate commitment
Connective tissue development
Wnt signaling
Respiratory system development
Ossification
Cardiac cell fate commitment
Cardiac muscle cell fate commitment
Chemical synaptic transmission
Epithelial to mesenchymal transition
Axongenesis
Synaptic transmission GABAergic
Anterior posterior axon guidance
Interneuron axon guidance
Neuron Axon guidance
Mesenchymal cell development
Mesenchymal stem cell differentiation
Forebrain neuron development
Noradrenergic neuron development
Bulb interneuron development
Response to immune
Response to stress
B
LuCaP 35CR
Neurogenesis & axonogenesis & Synaptic transmission
ASCL1
POU3F2
SOX2
FOXA2
CHGA
SYP
ENO2
NCAM1
GRIN2D
ADRA2A
CHRNA3
NRXN3
NLGN1
SYT4
TUBB2B
DRD2
NKX2-1
PROM1
SYT2
DRD3
CSCs & WNT & EMT
RUNX1
SOX9
SOX4
ZEB2
WNT5A
WNT5B
WNT3
WNT6
FZD6
FZD7
AXIN2
DKK1
DKK3
LEF1
CDH11
COL12A1
COL3A1
MMP2
MMP14
MMP24
DCN
DKK1
PROM1
TCF4
Log2FC
1.5
0
-1.5
C
Veh
LGK
Taz
Com
LuCaP 35CR
CD44
SOX2
Staining intensity(a.u.)
D
LuCaP 35CR
Cell cycle
Cell division
DNA replication
G1/S transition
G2/M transition
DNA Mitotic cell cycle
DNA recombination
Z score
E
LuCaP 35CR
MyC-CaP CR
Tumor mass (g)
Veh
Taz
LGK
Com
ns
F
LuCaP 35CR
MyC-CaP CR
Body weight (g)
Days
G
MyC-CaP CR
H3K4me3
H3K27me3
V
Taz
LGK
Com
-3
TSS
3kb
H
LuCaP 35CR
1kb
10kb
KMT2B
EZH2
EED
ASCL2
CHRNA7
TWSIT1

Supplemental Figure 8 Induction of tumor cell state diversity by PRC2/EZH2 inhibition can be mitigated by Wnt/β-catenin signaling blockade through altering chromatin bivalency.

A. Heatmaps of GSVA score of indicated programs detected by RNA-seq in MyC-CaP CR tumors in mice treated as indicated for 10 days.

B. Heatmaps display log_2 fold changes of gene expression detected by RNA-seq in LuCaP 35CR tumors in mice treated as indicated for 10 days.

C. Representative images of confocal microscopy analysis of indicated CSP protein expression in LuCaP 35CR tumors in mice treated as indicated (n = 5 mice per group). Scale bars, 50 μm. Note: the experiment here shares samples of Veh and Taz treatments with the one in Figure 2C and Supplemental Figure 2H. Bar graphs display ImageJ-analyzed immunofluorescence intensity from 7 randomly selected fields per tumor. a.u., arbitrary unit. **** $p < 0.0001$. Statistical significance determined by Student's *t* test.

Bar graphs display ImageJ-analyzed immunofluorescence intensity from 7 randomly selected fields per tumor. a.u., arbitrary unit. **** $p < 0.0001$.

D. Heatmaps of GSVA score of indicated programs detected by RNA-seq in LuCaP 35CR tumors in mice treated as indicated for 10 days.

E and F. Mouse tumor weight and picture (E) and mouse body weight (F) in mice treated by vehicle or indicated compounds for indicated days, 7 times per week, n = 5 mice per group. ** $p < 0.01$; *** $p < 0.001$.

G. Signal profiles of H3K4me3 and H3K27me3 ChIP-seq within +/- 3 kb windows around TSS at bivalent CSPs in MyC-CaP tumors in mice treated as indicated for 10 days.

H. IGV snapshots of EZH2, EED and KTM2B ChIP-seq peak at representative CSP genes in LuCaP 35CR tumors in mice treated as indicated for 10 days.
