# Supplementary material for: Targeting Wnt/β-catenin and circadian regulator restores PRC2/EZH2-controlled chromatin bivalency and suppresses cell state diversity
Source: J Clin Invest. 2026 Mar 17;136(9):e200260. doi: 10.1172/JCI200260 (PMC13132380; doi:10.1172/JCI200260)

Figure 4H. LNCaP cells were treated with indicated compounds and concentration (left panel)

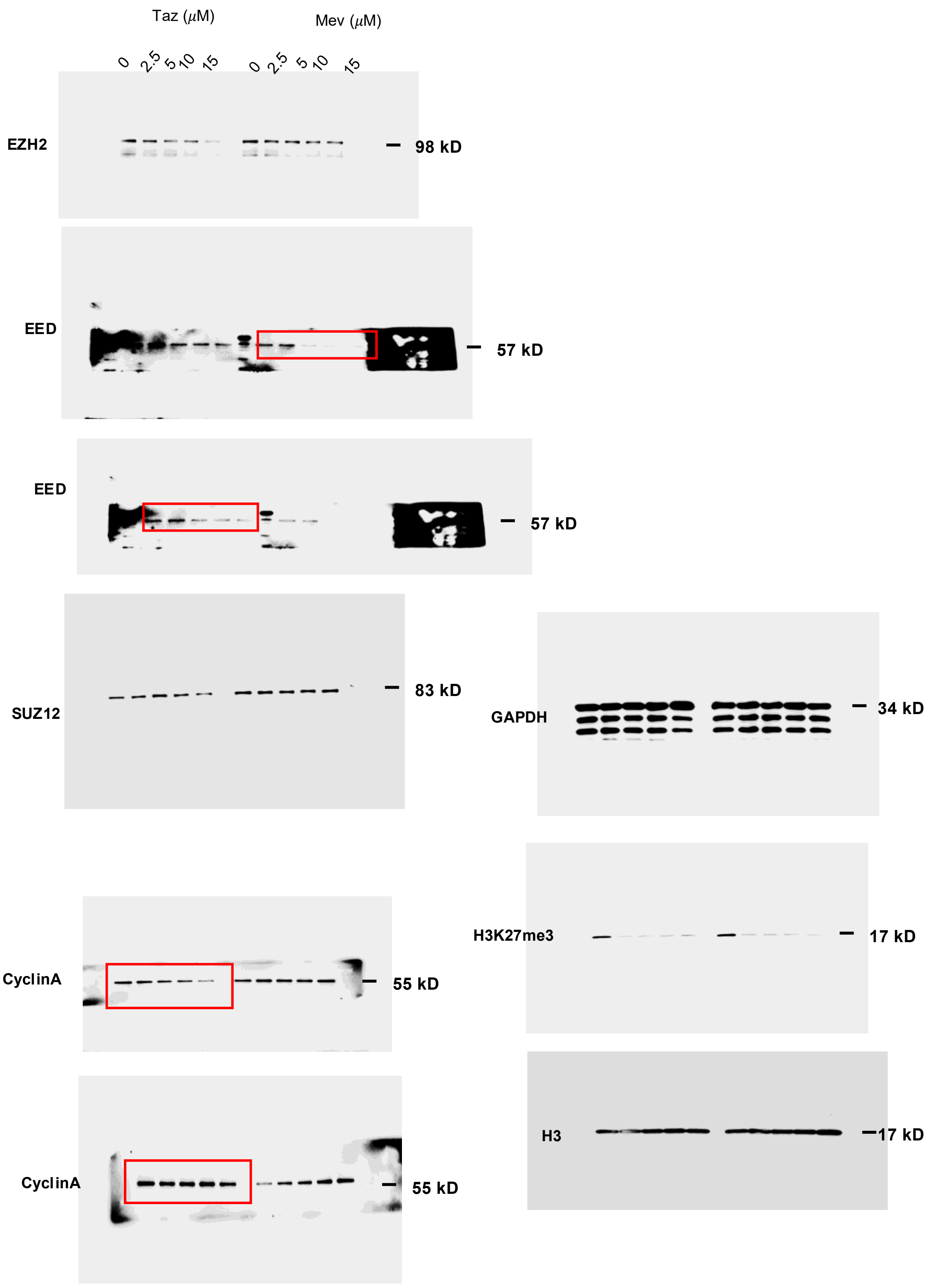

Figure 4H. LNCaP cells were treated with indicated compounds and concentration (middle panel)

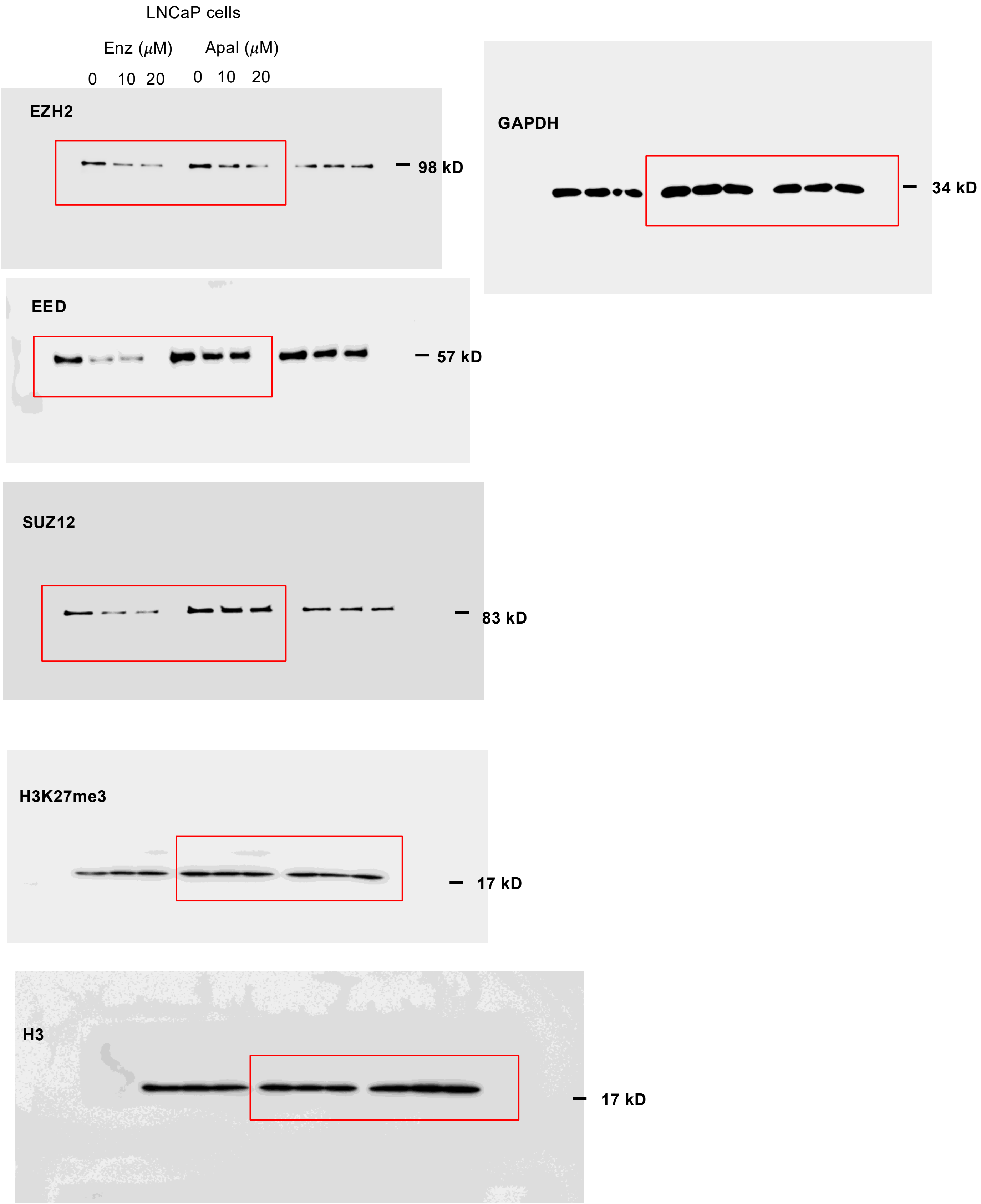

Figure 4H. LNCaP cells were treated with indicated compounds and concentration (right panel)

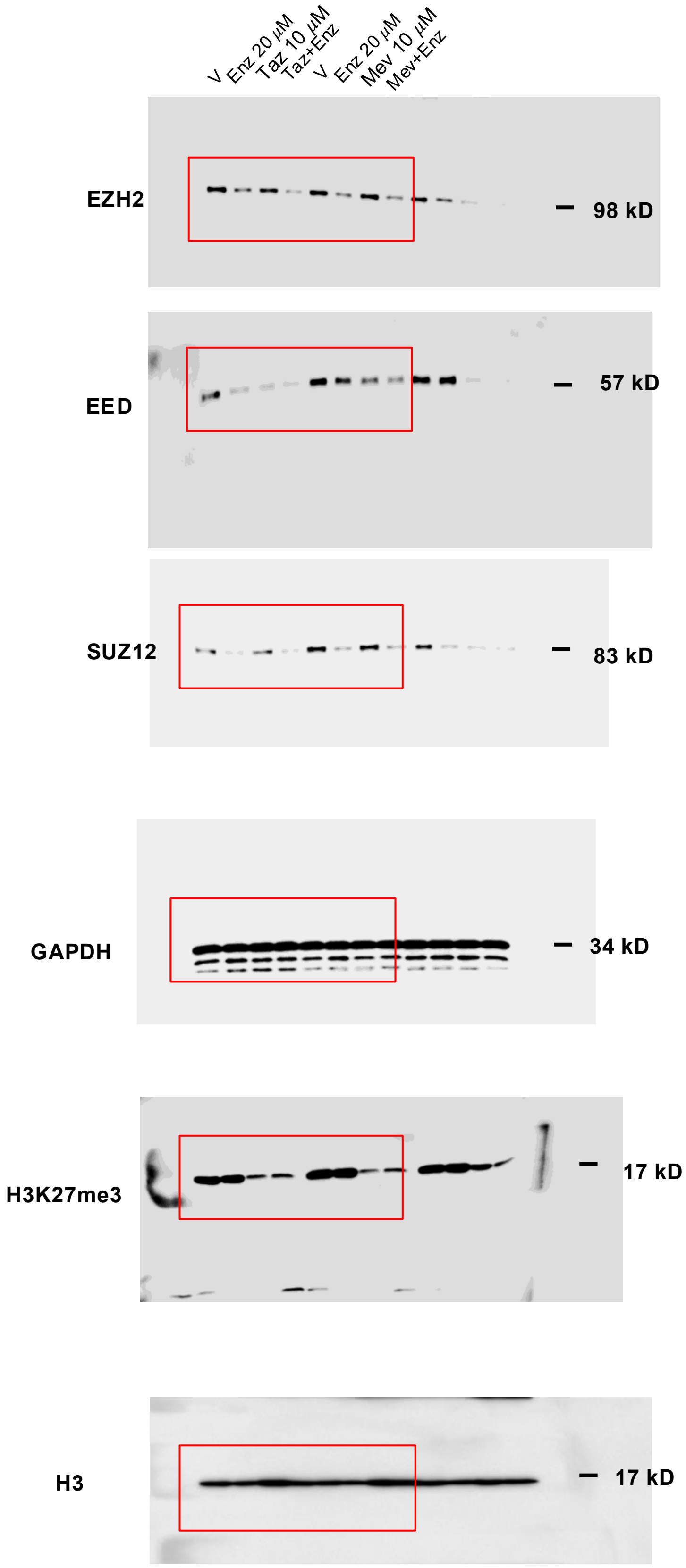

Supplemental Figure 4H. C4-2B and 22RV1 cells were treated with indicated compounds and concentration. （top-left panel,C4-2B cells）

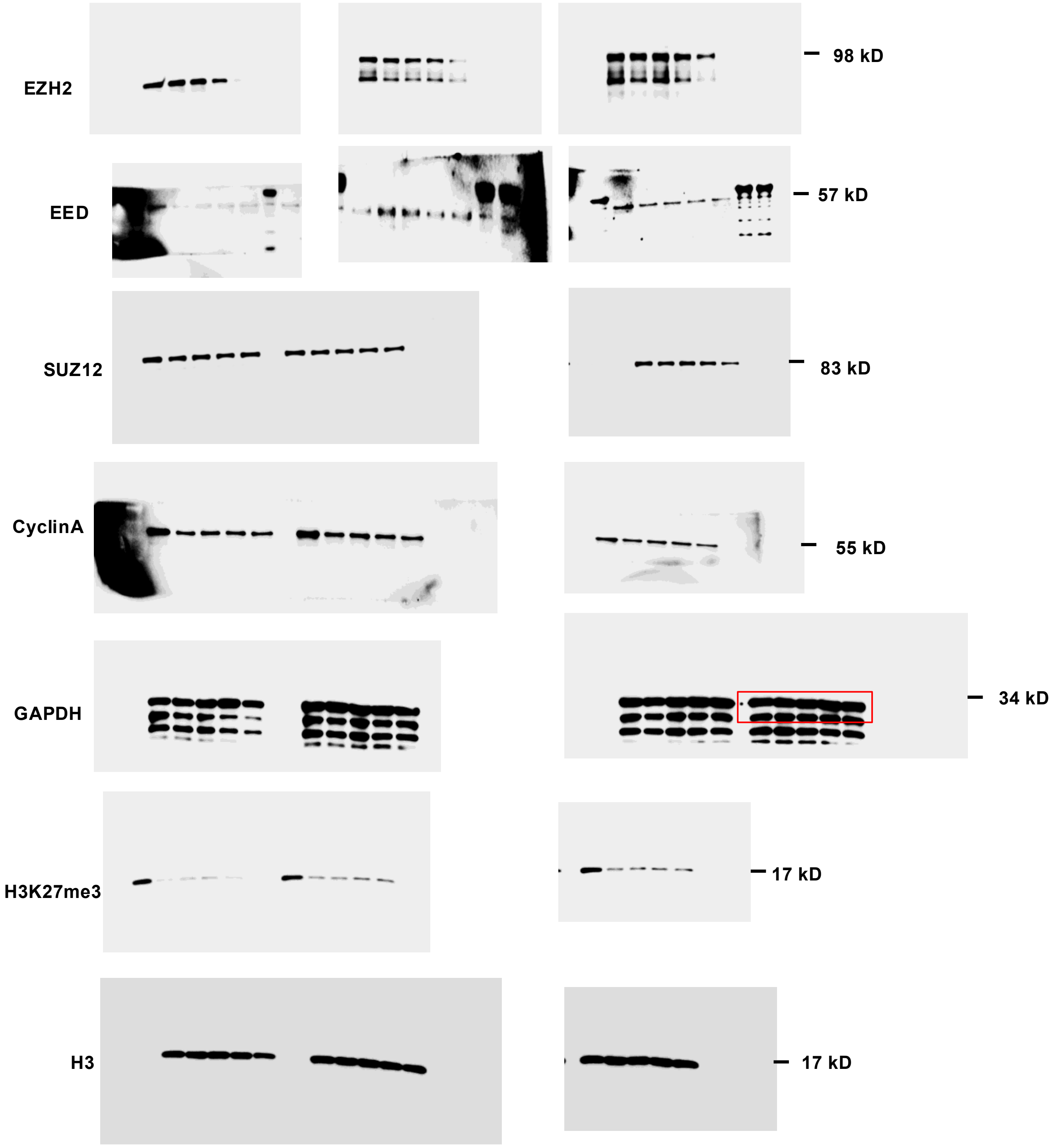

Supplemental Figure 4H. C4-2B and 22RV1 cells were treated with indicated compounds and concentration. （bottom-left panel, 22RV1 cells）

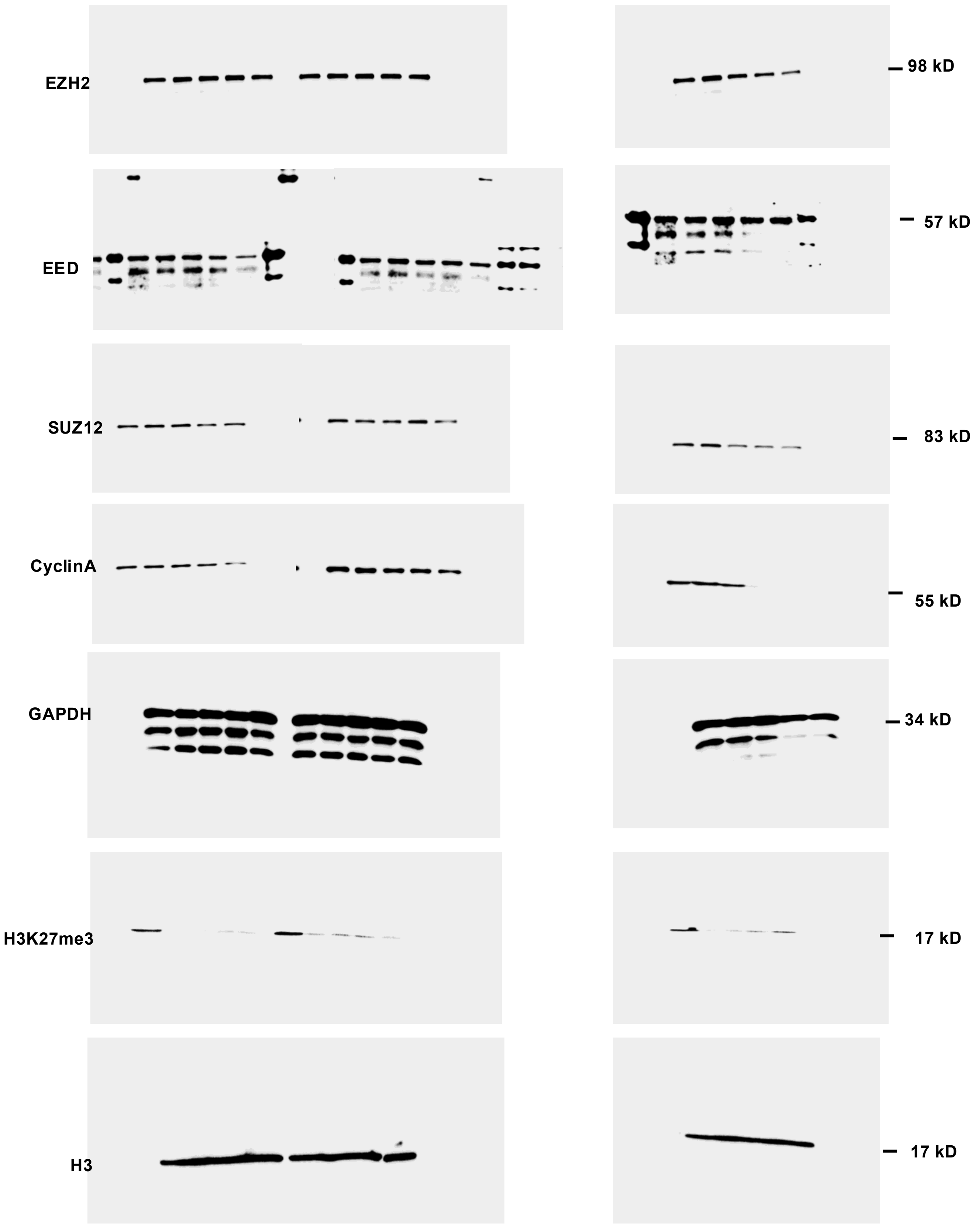

Supplemental Figure 4H. C4-2B and 22RV1 cells were treated with indicated compounds and concentration. （top-right panel）

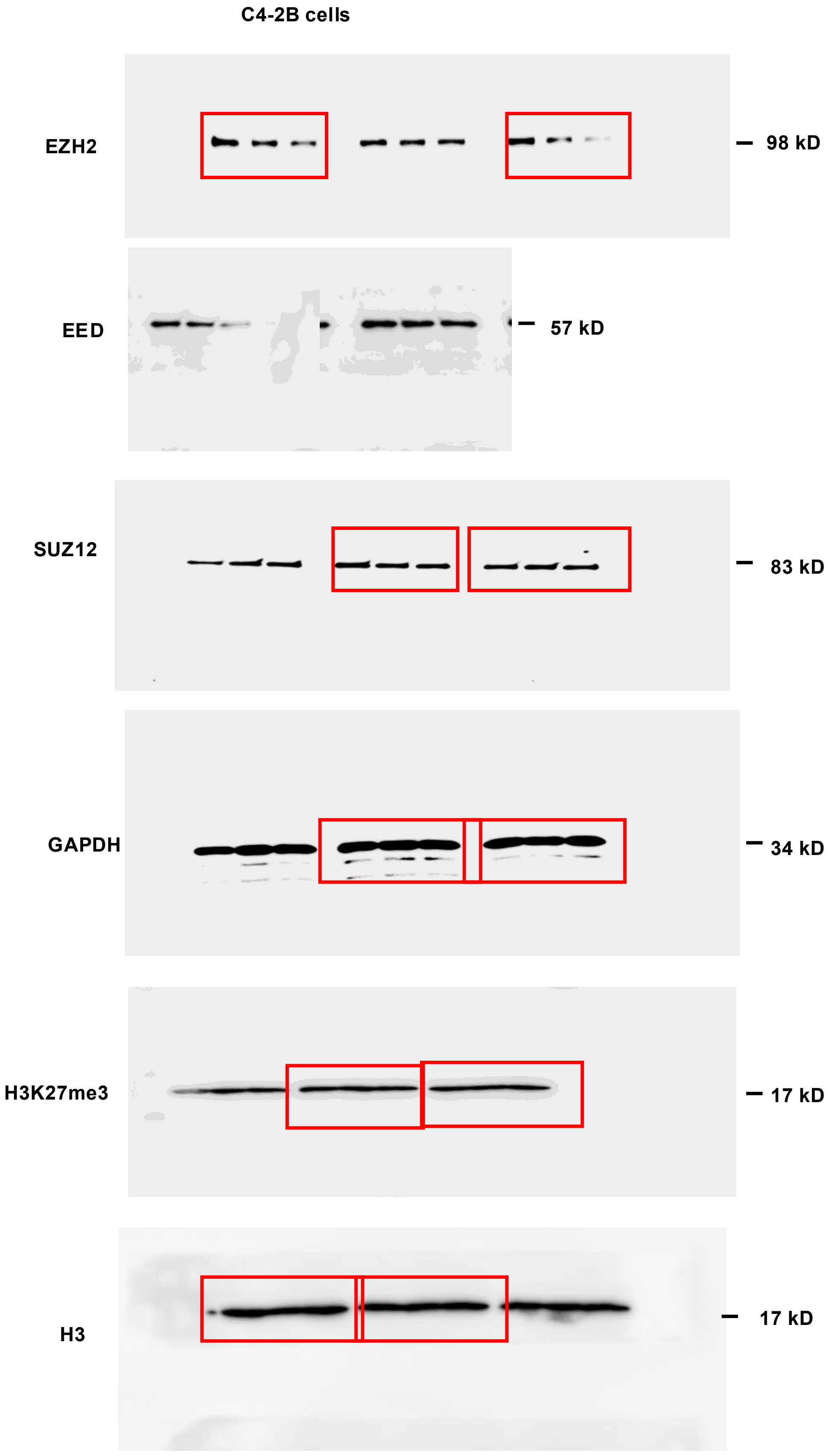

Supplemental Figure 4H. C4-2B and 22RV1 cells were treated with indicated compounds and concentration. (middle-right panel)

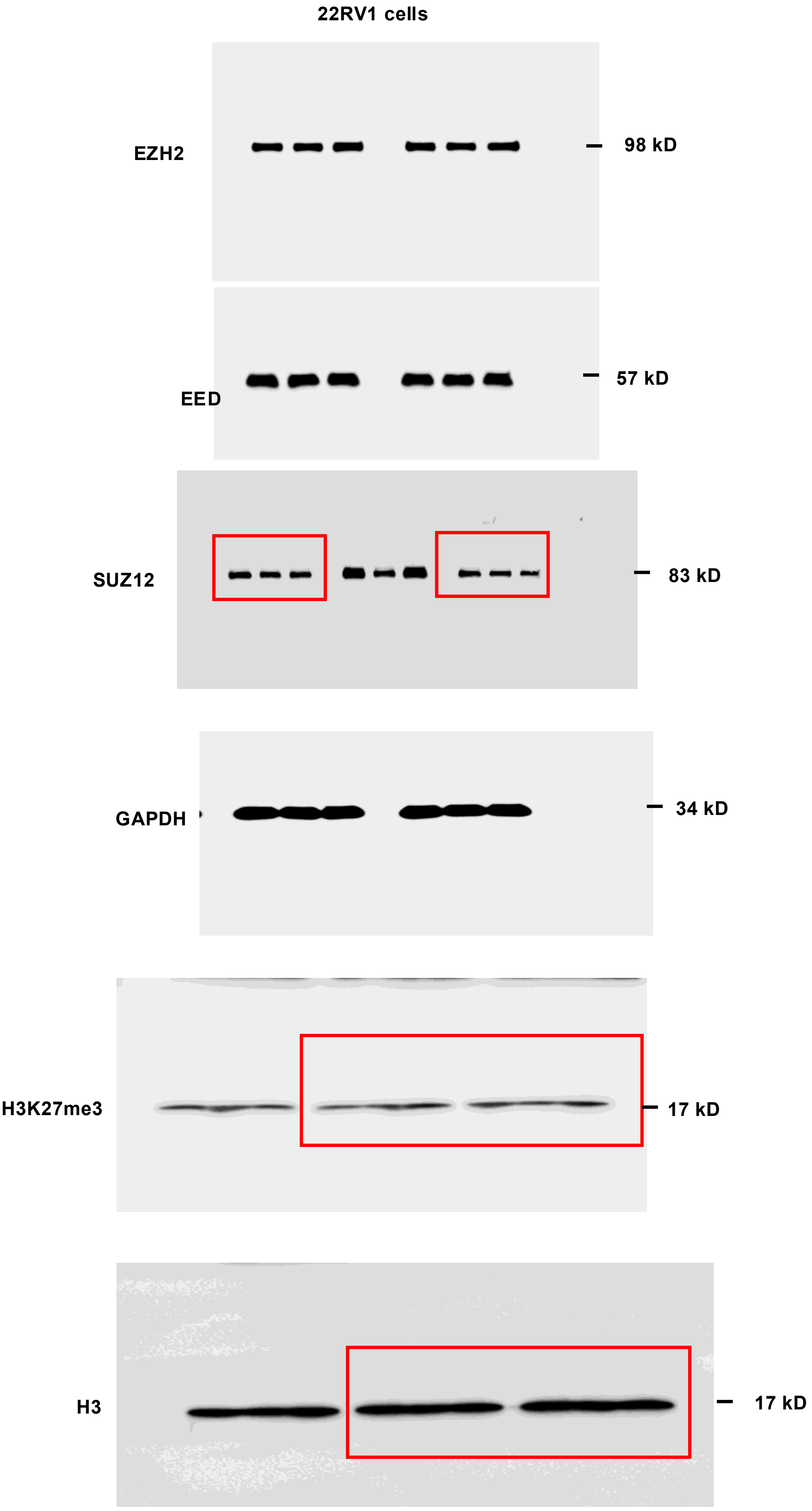

Supplemental Figure 4H. C4-2B and 22RV1 cells were treated with indicated compounds and concentration. （botom panels）

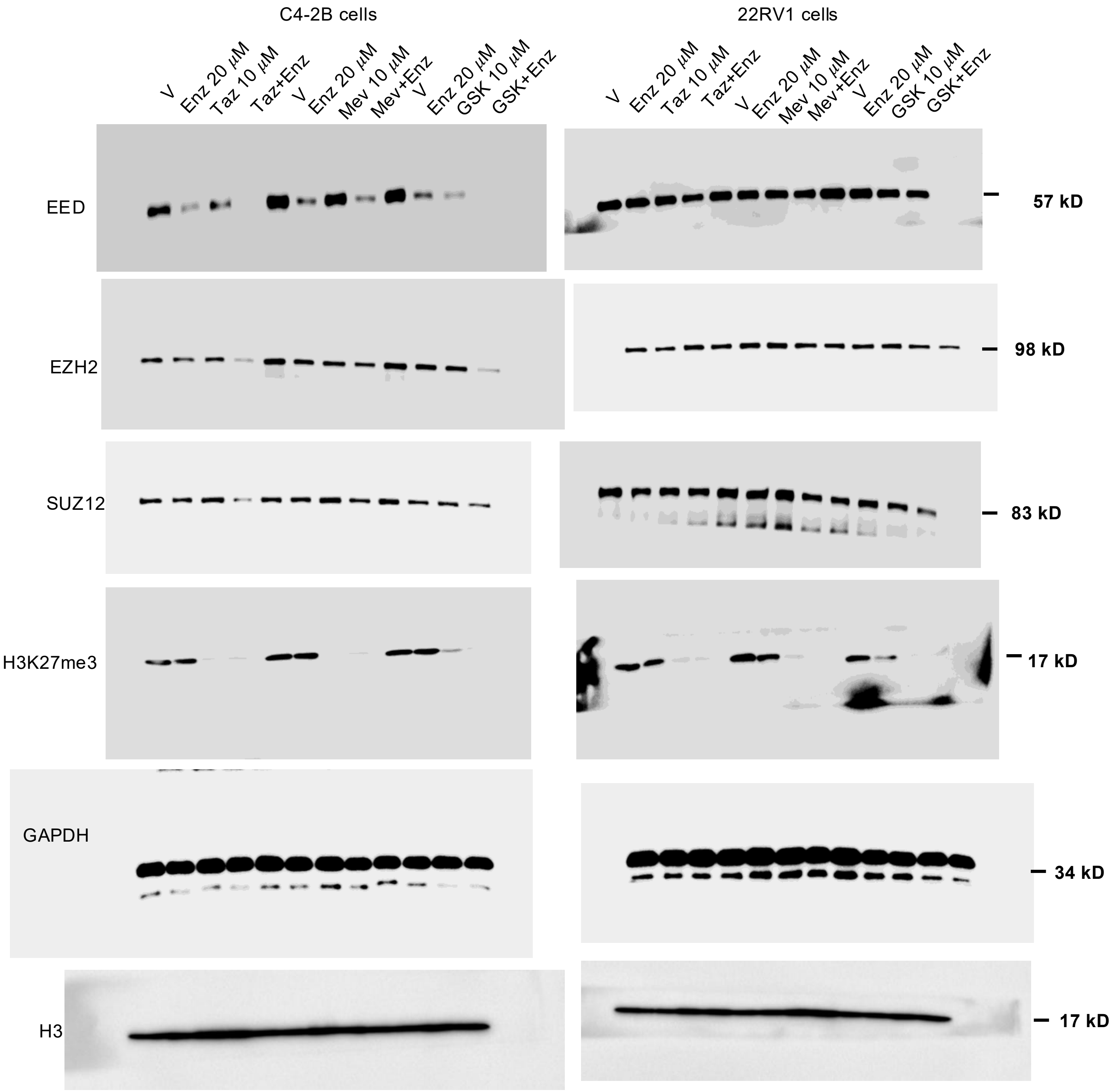

Supplement: Unedited blot and gel images [file jci-136-200260-s037.pdf]
